# Impact of Microwave Treatment on Protein and Starch Characteristics of Black Wheat and Whole Wheat (*T. aestivum* L.) Flour

**DOI:** 10.3390/foods15193537

**Published:** 2026-10-03

**Authors:** Alexandra Maria Uivarasan, Leonard Mihaly Cozmuta, Anca Peter, Camelia Nicula, Zsolt Szakács, Ersilia Alexa, Sylvestre Dossa, Marieta Muresan Pop, Klara Magyari, Monica Baia, Ioan Botiz, Lucian Barbu Tudoran, Zorica Vosgan, Beatrice Mihalescu, Cristina Pintilii, Lucica Maria Pop, Alexandru Babiciu, Monica Tarau, Adrian Cosma, Melisa Marcus, Anca Mihaly Cozmuta

**Affiliations:** 1 North University Center of Baia Mare, Technical University of Cluj Napoca, Victoriei Str. 76, 430122 Baia Mare, Romaniaszakacsz@yahoo.com (Z.S.); lucica_pop74@yahoo.it (L.M.P.);; 2 Faculty of Food Engineering, University of Life Sciences “King Michael I” from Timisoara, 300645 Timisoara, Romania; ersiliaalexa@usvt.ro (E.A.);; 3 Nanostructured Materials and Bio-Nano-Interfaces Center, Interdisciplinary Research Institute on Bio-Nano-Sciences, Babes-Bolyai University, T. Laurian 42, 400271 Cluj-Napoca, Romania; marieta.muresan@ubbcluj.ro (M.M.P.); klara.magyari@ubbcluj.ro (K.M.); ioan.botiz@ubbcluj.ro (I.B.); 4 INSPIRE Research Platform InfoBioNano4Health & Biomedical Imaging, Babes-Bolyai University, Arany Janos 11, 400084 Cluj-Napoca, Romania; 5 Faculty of Physics, Babes-Bolyai University, M. Kogălniceanu 1, 400347 Cluj-Napoca, Romania; 6 Faculty of Biology and Geology, Babes-Bolyai University, Clinicilor Str. 5-7, 400006 Cluj-Napoca, Romania; lucian.barbu@ubbcluj.ro; 7 National Institute for Research and Development of Isotopic and Molecular Technologies, Donath Str. 67-17 103, 400293 Cluj-Napoca, Romania

**Keywords:** black wheat flour, whole wheat flour, microwave processing, starch crystallinity, protein secondary structure, FTIR deconvolution, XRD analysis

## Abstract

This study investigated the structural and functional responses of whole wheat flour (WWF) and black wheat flour (BWF) to microwave (MW) dielectric heating. MW exposure impaired gluten network stability in WWF, whereas BWF exhibited limited cohesive gluten formation, acting as an elasticity-attenuating component. FTIR and XRD analyses demonstrated significant increases in relative starch crystallinity after 220 s of MW exposure, due to Vh-type complexation. AFM confirmed pronounced increases in surface roughness (BWF: S_a_ +39%, S_q_ +41%; WWF: S_a_ +42%, S_q_ +30%). Protein secondary structure deconvolution confirmed thermal denaturation rather than beneficial ordering: native β-sheets decreased (WWF: 37.04% to 32.68%; BWF: 37.97% to 31.49%), while β-turns increased (WWF: 22.32% to 30.14%). Water contact angle measurements showed reduced surface hydrophobicity (−19.20% in WWF, −22.70% in BWF). Thiol-disulfide (-SH/-SS) determinations highlighted different molecular mechanisms: WWF underwent intensive disulfide cross-linking (+419.6%), whereas BWF preserved free thiols (+75.5%) due to protective phenolic–protein complexation. Granular thermal damage increased in vitro enzymatic amylolysis, significantly elevating the amount of rapidly digestible starch (RDS) and estimated glycemic index (eGI). Overall, MW processing preferentially remodeled protein networks in WWF while predominantly altering starch matrix morphology in BWF, providing key insights into the dielectric thermal processing of conventional and pigmented wheat flours.

## 1. Introduction

For centuries, cereal-based foods have represented a cornerstone of the human diet, contributing to nearly 70% and about 50% of the daily caloric and total protein intake, respectively, while also supplying the essential macronutrients required for growth and maintenance [1]. Among cereals, wheat (*Triticum aestivum*) holds a central role due to its adaptability, storability, favorable nutritional profile, and, most importantly, to the unique viscoelastic properties of its dough, which distinguishes it from other cereals and underpins its technological significance in bread-making [2]. Structurally, wheat grains are composed of 10–15% proteins, 60–70% starch, and 3–8% non-starch polysaccharides [3].

Starch, the major carbohydrate fraction, occurs as semicrystalline granules composed mainly of amylose, a mostly linear α-(1-4)-linked glucan, and amylopectin, a highly branched polymer with α-(1-4) and α-(1-6) linkages. Amylopectin forms crystalline domains in polymorphic arrangements: A-type (cereals), B-type (tubers, legumes), and V-type, generated when amylose complexes with lipids [4]. These structures govern key functional properties such as gelatinization, retrogradation, and digestibility. Wheat proteins, primarily glutenins and gliadins, form the gluten network: gliadins confer extensibility, while glutenins, linked by disulfide bonds (–SS), provide elasticity and strength [5]. The stability of this network is influenced by secondary structural conformations (α-helix, β-sheet, β-turn, random coil) and by the balance of covalent (-SS) and non-covalent interactions [6].

Processing conditions strongly affect starch and protein organization in cereal matrices. Compared to conventional conducto-convective thermal methods (such as dry-air oven roasting or kilning), microwave (MW) dielectric heating offers operational and thermodynamic advantages. By directly coupling electromagnetic energy with endogenous dipolar water molecules, it delivers rapid volumetric heating that eliminates steep internal temperature gradients, uneven moisture distribution, and surface scorching [7,8]. Moreover, MW processing achieves targeted macromolecular reorganization within seconds, rather than hours, offering superior energy efficiency while better preserving heat-sensitive bioactive components (such as anthocyanins and phenolic acids in pigmented grains) from prolonged thermal degradation [9,10]. In starch, MW exposure modifies granule morphology, increases crystallinity degree, and alters gelatinization and retrogradation [7]. Proteins are also susceptible, with evidence of shifts from β-turn and random coil toward more ordered α-helix and β-sheet structures, supplemented by altered thiol-disulfide interchange [11,12]. These modifications can reshape dough rheology, water-binding, and digestibility.

Despite increasing attention to microwave (MW) effects on isolated starches and proteins, the behavior of complex whole flour matrices remains significantly underexplored. This gap is particularly evident for pigmented varieties such as black wheat. Unlike refined flour, black wheat flour is rich in anthocyanins and phenolic compounds capable of interacting with starch and proteins by non-covalent bonds. These bioactive constituents may competitively interfere with water distribution and biopolymer cross-linking, thereby modulating thermal stability and functionality [13,14]. However, the synergistic or antagonistic effects of MW treatment on starch–protein–phenolic interactions in black wheat flour (BWF) have not been systematically compared with conventional whole wheat flour (WWF).

To address this gap, this study provides a comprehensive investigation into the structural and functional responses of WWF and BWF to microwave exposure. By integrating rheological, spectroscopic, microscopic, and in vitro digestion analysis, this work offers insights into the molecular mechanisms underlying starch and protein reorganization. Furthermore, it elucidates how the unique phenolic profile of black wheat influences these transformations, establishing a basis for optimizing the technological and nutritional performance of functional cereal-based systems.

## 2. Materials and Methods

### 2.1. Chemicals and Reagents

All reagents used in this study were of analytical grade. Sodium hydroxide, hydrochloric acid, ethylenediaminotetraacetic acid (EDTA), nitric acid, trichloroacetic acid, TRIS [2-Amino-2-(hydroxymethyl) propane-1,3-diol], sodium dodecyl sulfate (SDS), n-hexane, cysteine, glycine, urea, β-mercaptoethanol, and Ellman’s reagent [5,5′-dithiobis-(2-nitrobenzoic acid), DTNB] were aquired from Merck (Darmstadt, Hesse, Germany).

### 2.2. Raw Materials

Whole wheat grain (WWF) was obtained from local agricultural producers in Maramureș County, Romania (harvest year 2024; cultivar *Triticum aestivum* cv. Lovrin), cultivated under conventional, rainfed agricultural management in a temperate continental climate. Black wheat grain (BWF) was sourced from Glorious Inheriting Store (Wuhan, China) (North Asia; harvest year 2024; pigmented *Triticum aestivum* L. genotype) from commercial production under standard conventional farming practices. Both grain lots complied with standard milling quality specifications (intact, non-germinated kernels, foreign matter < 1%). Prior to processing, grains were cleaned, conditioned to 14% moisture, and stored in airtight containers in a cool, dry, and well-ventilated environment at room temperature (20 ± 2 °C, relative humidity < 60%) to prevent moisture fluctuations. Commercial refined white wheat flour (Type 550/BL 55; Gyermelyi Zrt., Gyermely, Hungary) was purchased from a local market and used as the standard carrier flour for dough rheological characterization in Section 3.1. The baseline composition of the carrier white wheat flour consisted of 13.7% moisture, 10.0% crude protein (as is basis, corresponding to 11.6% dry basis; N × 5.7), 0.53% ash (dry basis), and 1.0% crude fat. Native and microwave-treated whole wheat flour (WWF) and black wheat flour (BWF) were systematically blended into this standard white carrier base at substitution levels of 10% and 20% (*w*/*w*) to assess their techno-functional mixing and pasting behavior. The comprehensive proximate analysis (protein, crude fat, ash, moisture), individual mineral profile, and phenolic composition (total free phenolics, flavonoids, HPLC individual polyphenols, and DPPH antioxidant capacity) of the native WWF and BWF flours were determined as part of our foundational baseline investigation and are reported in detail elsewhere [15]. A summary of these baseline compositional and phytochemical characteristics is provided in Table S1 (Supplementary Material) to support the interpretation of structural and rheological modifications.

### 2.3. Microwave Treatment of Wheat Flour Samples

Microwave treatment of whole and black wheat flour samples was performed using a domestic microwave oven (Samsung, Surrey, UK) equipped with a ceramic interior cavity and a 23 L chamber volume and operated at a fixed power of 800 W. Precisely weighed 5 g portions of flour, obtained from freshly milled grains, were subjected to thermal treatment in their native state (baseline moisture content: 8.40 ± 0.07% for WWF and 11.36 ± 0.13% for BWF; Table S1) without prior moisture standardization. This native moisture processing was purposefully adopted to replicate real-world post-milling industrial stabilization practices and to prevent premature activation of endogenous enzymes or matrix plasticization prior to irradiation.

To ensure uniform field exposure, each flour sample was evenly spread in a circular monolayer of approximately 1 mm thickness on a flat ceramic plate and systematically positioned at a constant radial distance on the rotating turntable. Samples were irradiated across designated exposure intervals ranging from 0 to 220 s (at 20 or 40 s increases). To document the precise thermal history and assess whether initial moisture differences influenced dielectric heating kinetics, sample temperatures were recorded immediately upon treatment ending at each milestone using an infrared sensor (Table S2). Both flour samples exhibited parallel heating rates, converging at equivalent terminal temperatures of 152.1 ± 3.5 °C (WWF) and 150.6 ± 3.2 °C (BWF) at 200 s, thereby confirming equivalent thermal input across treatments. Following irradiation, samples were rapidly homogenized, transferred into 45 mL airtight tubes, and stored in the dark at 8 °C to prevent moisture uptake or retrogradation until further analysis.

### 2.4. Rheological Analysis (Mixolab)

Rheological analysis was performed using a Chopin Mixolab (Chopin Technologies, Paris, France) following the ‘Chopin+’ protocol [16]. Flour samples (42–50 g, depending on moisture content) were placed in the Mixolab bowl and mixed. After tempering, water was added to achieve optimal dough consistency, with particular attention given to water absorption to ensure complete hydration of all components.

The evaluated Mixolab profile parameters were: water absorption, dough development time, dough stability (resistance to mixing), maximum torque during mixing (C1), protein weakening (C2) induced by mechanical stress and heat, starch gelatinization rate (C3), minimum torque (C4), and torque after cooling to 50 °C (C5). Additional calculated parameters included cooking stability (C4/C3), protein weakening under heating (α slope), starch gelatinization rate (β slope), enzymatic degradation rate (γ slope), and starch retrogradation during cooling (C5–C4) (Supplementary Material), the latter being indicative of the storage stability of the final products [13].

### 2.5. Starch Isolation and Characterization

Primary starch was isolated from wheat flour using the dough-ball method, according to the protocol described by Pauly et al. [17]. Briefly, 25 g of flour (12% moisture content) was mixed with 13.45 mL of deionized water to form an optimal dough. Starch was then washed out from the dough with deionized water (approximately 500 mL) until the wash water became clear. The resulting starch suspensions were further purified by sieving (38 μm mesh; Retsch, Haan, Germany) followed by centrifugation (5800 rpm, 10 min), after which the supernatants were discarded. The starch pellets were washed three times with deionized water (~400 mL each), with supernatants removed after each washing step. The superficial starch layer of the pellets was carefully removed using a spatula, and the primary starch fractions (the lower pellet layers) were collected. These fractions were air-dried at room temperature prior to subsequent analyses.

#### 2.5.1. FTIR Analysis

FTIR absorbance spectra within the range of 4000–600 cm^−1^ were obtained using a Perkin Elmer BX2 spectrometer (Waltham, MA, USA) equipped with a Pike Miracle diamond ATR crystal (PerkinElmer, Waltham, MA, USA). Dried flour samples were placed directly on the crystal and scanned in ten replicates at a resolution of 1 cm^−1^ under constant pressure, resulting in ten independent spectra per sample. To reduce background noise, radiation scattering, and intrinsic fluorescence, the spectra were corrected, normalized to the 0–1 range, and processed using a Savitzky–Golay filter (polynomial order = 3, window size = 9). This procedure ensured that the observed absorbance variations reflected only the changes in the chemical composition of the samples.

The short-range molecular order index of starch was determined from the deconvoluted FTIR spectra by assigning the absorption bands at 1047 cm^−1^ and 995 cm^−1^ to crystalline structures and the band at 1020 cm^−1^ to the amorphous fraction [18]. The ratio of the integrated areas of crystalline-associated bands to amorphous-associated bands was then used to calculate the FTIR-derived order index (1047/1020 cm^−1^). Protein secondary structures were quantified by evaluating the relative contributions of β-sheets (1636–1615 cm^−1^ for high frequency; 1700–1682 cm^−1^ for low frequency), random coils (1645–1637 cm^−1^), α-helices (1663–1646 cm^−1^), and β-turns (1681–1664 cm^−1^) (Supplementary Material) [19,20].

#### 2.5.2. Atomic Force Microscopy (AFM)

AFM imaging was performed in non-contact (tapping) mode using a system from Molecular Devices and Tools for Nano Technology (NT-MDT, Moscow, Russia), mounted on an Olympus IX71 optical microscope supplied by Spectrum Instruments Ltd. (Limerick, Ireland). High-resolution Noncontact Golden Silicon probes (NT-MDT) were employed, characterized by a tip radius of less than 10 nm and a height of 14–16 µm. Each probe was gold-coated on the cantilever surface facing the detector. The cantilever had a length of 125 ± 5 µm, a resonance frequency of 187–230 kHz, and a nominal force constant ranging from 1.45 to 15.1 N/m. Images (256 × 256 lines) were recorded at a scanning speed of 1–2 µm/s, with the setpoint adjusted between 9 and 12 V to ensure an exceptionally gentle tapping regime.

#### 2.5.3. X-Ray Diffraction (XRD)

The long-range ordered crystalline structure and phase transitions of the samples were analyzed using a Shimadzu X-ray diffractometer (XRD 6000, Kyoto, Japan) using the Cu Kα radiation (λ = 1.54 Å) and a Ni filter. Diffraction patterns were collected over a 2θ range of 10–80° at a scanning rate of 2°/min.

#### 2.5.4. Scanning Electron Microscopy (SEM)

The morphology of starch granules was examined using scanning electron microscopy (SEM) with a dual-beam FEI Quanta 3D FEG microscope operated under high-vacuum conditions. The instrument was equipped with an Everhart–Thornley detector (ETD) for secondary electron imaging and operated at an accelerating voltage of 30 kV, providing the resolution required to observe fine structural details of the samples.

#### 2.5.5. In Vitro Starch Digestion Kinetics and Nutritional Fractions

In vitro starch digestion kinetics was determined to simulate gastrointestinal hydrolysis following the protocol described by Goñi et al. [21], with minor modifications. Briefly, 200 mg of flour was suspended in 10 mL of sodium acetate buffer (0.1 M, pH 5.2, containing CaCl_2_) and pre-incubated at 37 °C for 10 min. The enzymatic hydrolysis reaction was initiated by adding 2.0 mL of a freshly prepared enzyme solution containing porcine pancreatin (α-amylase) and amyloglucosidase.

The reaction mixture was incubated at 37 °C under continuous agitation. Aliquots of 0.5 mL were collected at specific time intervals (0, 10, 20, 30, 60, 90, 120, and 180 min). Immediate enzymatic inactivation was achieved by transferring each aliquot into a test tube containing 2.0 mL of absolute ethanol. Following centrifugation (3000 rpm, 5 min), the concentration of reducing sugars (glucose equivalent) released into the supernatant was quantified spectrophotometrically at 540 nm using the 3,5-dinitrosalicylic acid (DNS) reagent according to Miller [22]. Absorbance values were converted to glucose concentration using a pre-established D-glucose standard calibration curve (R^2^ = 0.9962).

Starch nutritional fractions were calculated according to Englyst et al. [23] using the following equations:

(1) $$\mathrm{RDS}\left(\%\right)=\frac{G_{20}\times 0.9}{\mathrm{TS}}\times 100$$

(2) $$\mathrm{SDS}\left(\%\right)=\frac{\left(G_{120}-G_{20}\right)\times 0.9}{\mathrm{TS}}\times 100$$

RS (%) = 100 − (RDS + SDS)(3)

where:

RDS—rapidly digestible starch (hydrolyzed within the first 20 min); SDS—slowly digestible starch (hydrolyzed between 20 and 120 min); RS—resistant starch (unhydrolyzed after 120 min); G_0_, G_20_, and G_120_—the amounts of free glucose (mg) measured at 0, 20, and 120 min of enzymatic digestion, respectively; TS—the total starch content of the sample (mg), determined using a two-stage enzymatic hydrolysis followed by spectrophotometric quantification with 3,5-dinitrosalicylic acid (DNS) according to AACC Method 76-11.01 [24] adapted from Miller [19]; 0.9—the stoichiometric factor converting free glucose to anhydroglucose starch polymers.

Mathematical modeling of starch hydrolysis kinetics was performed in OriginPro software v 9.0 (OriginLab Corp., Northampton, MA, USA) by non-linear curve fitting using the first-order kinetic model established by Goñi et al. [18]:

*C_t_* = *C*_∞_ ⋅ (1 − *e*^−*k*⋅*t*^) (4)

where C_t_ represents the percentage of starch hydrolyzed at digestion time t (min), *C*_∞_ is the estimated equilibrium concentration of digestible starch (%), and k represents the kinetic rate constant (min^−1^).

The area under the hydrolysis curve (AUC, %⋅min) was calculated by analytical integration over the digestion window (0–180 min) based on the estimated kinetic parameters:

(5) $$\mathrm{AUC}=C_{\infty }\left(t_{f}-t_{0}\right)-\frac{C_{\infty }}{k}\left(1-e^{-k\left(t_{f}-t_{0}\right)}\right)$$

where *t_f_* is the final incubation time (180 min) and *t*_0_ is the initial time (0 min).

The hydrolysis index (HI, %) was calculated as the percentage ratio between the AUC of the analyzed flour sample and the AUC of the corresponding native reference flour (0 s = 100%):

(6) $$\mathrm{HI}=\left(\frac{{\mathrm{AUC}}_{\mathrm{sample}}}{{\mathrm{AUC}}_{\mathrm{reference}}}\right)\times 100$$

The estimated glycemic index (eGI) was subsequently derived using the equation proposed by Goñi et al. [18]:

eGI = 39.71 + 0.549 × HI(7)

### 2.6. Isolation and Characterization of Protein

Wheat flour was defatted according to the protocol described by Zhao et al. [19], with minor modifications. Flour samples were mixed with hexane (1:5, *w*/*v*), under continuous stirring at 200 rpm for 10 min, and centrifuged at 2500× *g* for 5 min. The supernatant was discarded, and the solid phase was remixed with hexane at the same ratio and centrifuged again. This extraction step was repeated three times, after which the defatted flour samples were dried on filter paper for 12 h.

Subsequently, the flour samples were sieved through a 0.25 mm mesh and suspended in a 0.1 M NaOH solution at a ratio of 1:9 (*w*/*v*). The mixture was stirred at 25 °C for 4 h then centrifuged at 6000× *g* for 10 min at 4 °C. The supernatant was adjusted to pH 5.5 using 2 M HCl to precipitate the proteins, followed by centrifugation for 10 min at 2500 rpm. The resulting protein precipitate was washed twice with distilled water, and the final pH of the suspension was adjusted to 7.0 with NaOH solution to achieve neutral conditions. The protein isolate was spread onto glass slides and air-dried for 24 h at 21 °C [19].

#### 2.6.1. Determination of Protein Isolate Surface Hydrophobicity

The evolution of surface hydrophobicity in wheat protein isolate during microwave treatment was assessed using the sessile drop method [25]. Protein isolate samples were evenly distributed on glass slides, and a 5 μL droplet of distilled water was dropped onto the surface of each sample. Images were captured using a Canon EF 100 mm f/2.8L Macro IS USM lens (Canon Inc., Tokyo, Japan), operated at maximum magnification (1×) and a high depth of field (f/13). Focusing was achieved by adjusting the sample position on the dedicated microscope stage. No white balance adjustments or other image post-processing was necessary beyond the camera’s settings.

Contact angles were measured using the ImageJ software (version 1.54, NIH, USA), using a dedicated angle-measurement plugin. For each droplet, the left and right contact angles were measured, and their mean value was calculated. All measurements were carried out within 15 s of droplet deposition to avoid water absorption by the protein matrix. The results for each flour sample were expressed as the average of contact angles measured across five independent replicates.

#### 2.6.2. Quantification of Free Sulfhydryl (-SH) Content

The free sulfhydryl (-SH) content of flour samples was determined following the method of Jiang et al. [26], with minor modifications. Briefly, 200 mg of protein isolate was mixed with 8 mL of buffer solution containing 2.5% sodium dodecyl sulfate (SDS), 4.0 mM ethylenediaminetetraacetic acid (EDTA), 92 mM glycine, and 86 mM Tris-HCl at pH 8.0. The mixture was vortexed for 1 h, after which 8 mL of Ellman’s reagent was added. Then, the samples were incubated at 25 °C for 30 min in the dark. Following centrifugation (6000 rpm, 10 min), the absorbance of the supernatant was measured at 412 nm using a UV–VIS spectrophotometer (Perkin Elmer Lambda 35) against a reagent blank. Since the protein isolates obtained from pigmented wheat samples display intrinsic coloration that could interact with Ellman’s reagent (DTNB) and alter the color of the final solution, a parallel control was prepared for each isolate by replacing DTNB with buffer. The absorbance of the control was subtracted from that of the corresponding sample, ensuring that the measured values were due only to the absorbance of the protein isolate, without interference from phenolic compounds. The free –SH content was calculated using a cysteine standard calibration curve (R^2^ = 0.9966) and expressed as μmol/g.

#### 2.6.3. Quantification of Disulfide (-SS) Bond Content

The disulfide bond (–SS) content was determined according to the protocol described by Liu et al. [7], with slight modifications. Precisely weighed protein-isolate samples (300 mg) were dispersed in 12 mL of Tris-Gly-8 M urea buffer (pH 8.0) and vortexed at room temperature for 1 h, followed by centrifugation at 6000 rpm for 10 min at 4 °C. A 3 mL aliquot of the resulting supernatant was then mixed with 0.3 mL of β-mercaptoethanol, vortexed in the dark at room temperature for 1 h, and subsequently combined with 12 mL of acetone pre-cooled to −20 °C. After an additional 1 h reaction at 4 °C, the mixture was centrifuged at 6000 rpm for 20 min at 4 °C.

The precipitate was washed with 15 mL of cold acetone and centrifuged to remove residual β-mercaptoethanol, followed by two additional washing steps. The final pellet was dissolved in 10 mL of Tris-Gly-8 M urea buffer, after which 10 mL of Ellman’s reagent (DTNB, 4 mg/mL) was added. The reaction was performed in the dark at room temperature for 30 min, then the solution was centrifuged at 6000 rpm for 5 min. Absorbance was measured at 412 nm, using an UV-VIS spectrophotometer (Perkin Elmer Lambda 35), against a blank consisting of Tris-Gly-8 M urea buffer and Ellman’s reagent (1:1, *v*/*v*). Control samples were not required in this case, as the absorbance values of the controls were negative. The total -SH content of the sample was determined from a cysteine standard calibration curve (R^2^ = 0.9990). The disulfide bond (–SS) content was subsequently calculated using Equation (8):

–*SS* (μM/g) = (*Total*_-*SH*_ − *Free*_-*SH*_)/2 (8)

where Total_-SH_ represents the total sulfhydryl content and Free-_SH_ the free sulfhydryl content.

### 2.7. Statistical Analysis

All experimental analyses were conducted in at least three replicates, and the results are expressed as mean values ± standard deviations (SDs). Differences between group means were assessed by one-way analysis of variance (ANOVA), followed by Tukey’s post hoc test for multiple comparisons, with a statistical significance set at *p* < 0.05. Pearson’s correlation coefficients (r) were calculated to assess the strength and direction of linear relationships among key variables, with statistical significance defined as *p* < 0.05.

Principal component analysis (PCA) was applied to explore multivariate relationships among pigmented wheat flour properties, including FTIR-derived starch order index, protein secondary structure, X-ray diffraction, in vitro starch digestibility and microwave treatment parameters. PCA transformed the original variables into orthogonal principal components, with PC1 accounting for the largest proportion of total variance and subsequent components (PC2, PC3, etc.) capturing progressively smaller fractions. Statistical analyses were performed using SPSS Statistics v.24 (IBM Corp., Armonk, NY, USA) and OriginPro v 9.0 (OriginLab Corp., Northampton, MA, USA).

## 3. Results and Discussion

### 3.1. Rheological Analysis of Wheat Flour Samples (Mixolab)

Mixolab rheological analysis was conducted to evaluate the thermomechanical behavior of whole wheat flour (WWF) and black wheat flour (BWF) composites, in both untreated and microwave-treated states, using a standardized water absorption of 60%. Due to different physical and compositional limits, pure flours (100%) and composite formulations were evaluated according to their technological dough-forming feasibility. Control whole wheat flour (WWF 0, 100%) formed a cohesive dough and was characterized to establish an external native whole-grain reference benchmark. In contrast, 100% BWF failed to generate a measurable Mixolab curve due to its inherently weak storage protein architecture and low gluten index. Similarly, pure MW-treated whole wheat flour (WWF 220) was unable to form a single workable dough due to extensive thermal protein denaturation. Consequently, to reflect practical baking fortification and ensure instrumental feasibility, both flours were incorporated into a standardized commercial white wheat flour carrier at 10% and 20% substitution levels, establishing a strictly controlled comparative framework within an identical carrier matrix. Importantly, this empirical framework does not evaluate the standalone rheology of pure treated flours; rather, it quantifies an interference and dilution effect, determining how microwave-modified biopolymer matrices affect the network development and hydration kinetics of a host gluten system.

**Figure 1:**
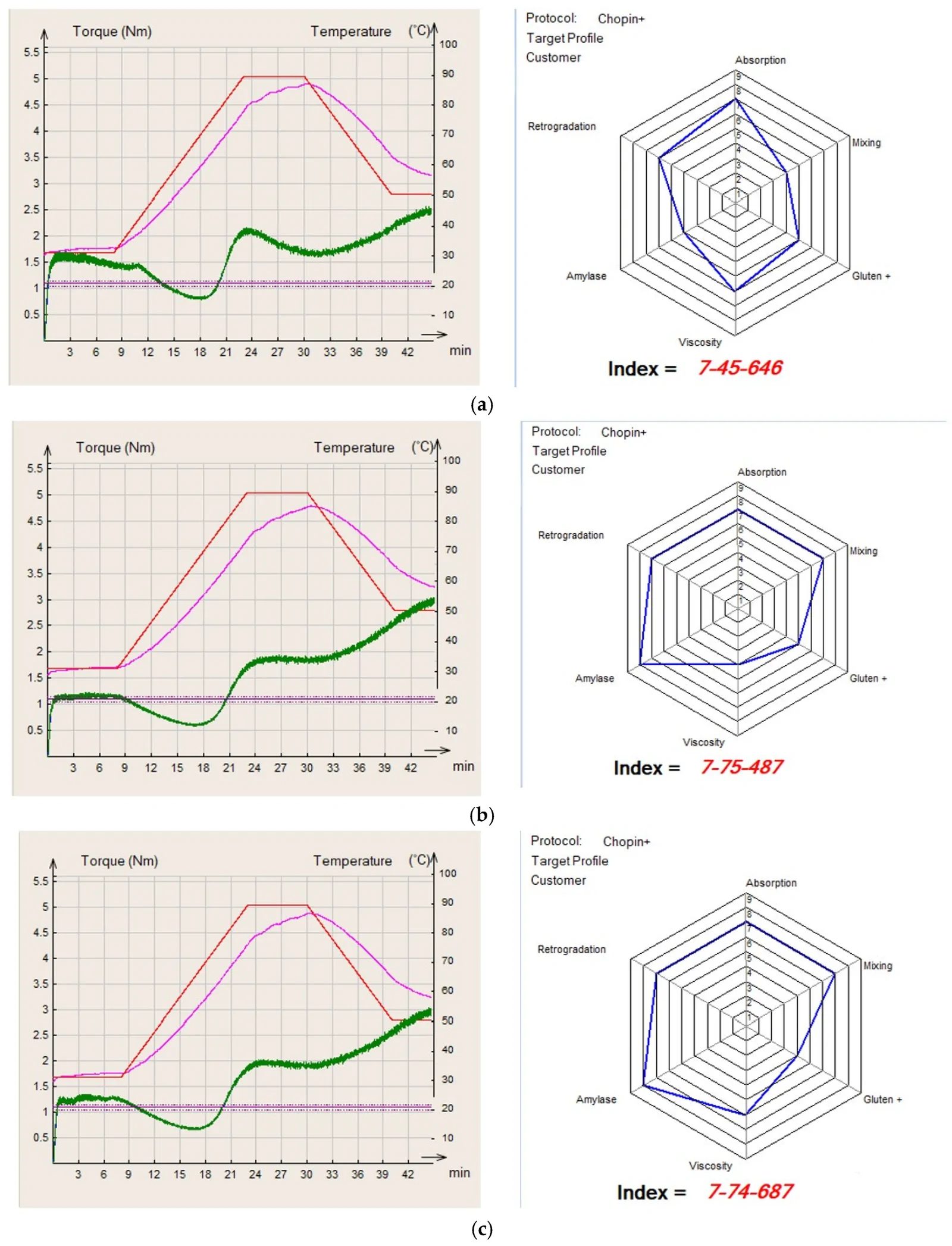

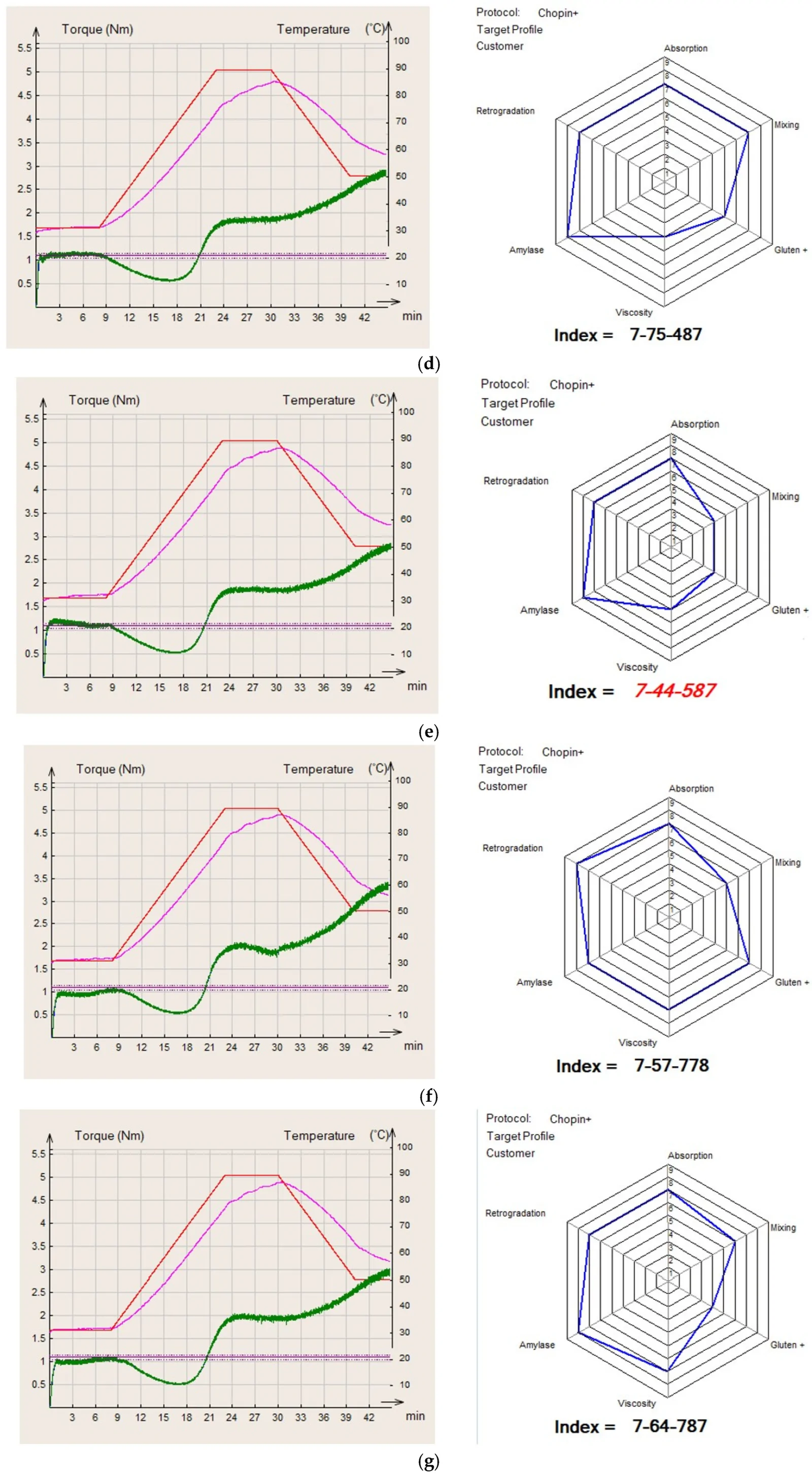
Mixolab rheological profiles of wheat flour samples: (**a**) WWF 0 (100%)—whole wheat flour not subjected to microwave treatment; (**b**) WWF 220 (10%)—mix of whole wheat flour (10%) treated with microwave irradiation for 220 s and white wheat flour (90%); (**c**) WWF 220 (20%)—mix of whole wheat flour (20%) treated with microwave irradiation for 220 s and white wheat flour (80%); (**d**) BWF 0 (10%)—mix of black wheat flour (10%) not subjected to microwave treatment and white wheat flour (90%); (**e**) BWF 0 (20%)—mix of black wheat flour (20%) not subjected to microwave treatment and white wheat flour (80%); (**f**) BWF 220 (10%)—mix of black wheat flour (10%) treated with microwave irradiation for 220 s and white wheat flour (90%); (**g**) BWF 220 (20%)—mix of black wheat flour (20%) treated with microwave irradiation for 220 s and white wheat flour (80%).

The torque values (C1–C5) and dough stability provide essential insights into the dynamics of protein–starch interactions during thermal processing: C1 indicates dough development and mixing tolerance; C2 reflects protein stability and susceptibility to thermal weakening; C3 corresponds to starch gelatinization capacity; C4 corresponds to hot-paste stability and cooking resistance under continuous shear (reflecting gel integrity following thermal enzyme inactivation; C5 provides information on starch retrogradation during cooling [27]. Figure 1a–g on the left displays the Mixolab curves obtained for the analyzed flour samples, representing the evolution of torque (Nm) and temperature (°C) over time (minutes). The red and blue lines correspond to block and dough temperatures, respectively, while the green line indicates dough resistance [13]. The figures on the right present the associated quality indices.

As illustrated in Figure 1a–g and Table 1, the control wheat flour WWF 0 (100%) (untreated) exhibited robust dough stability (10.40 min) along with high C1 (1.628 Nm) and C2 (0.816 Nm) values, characteristic of an intact, cohesive gluten network. Within the composite dough formulations, experimental effects were systematically evaluated across two primary comparative axes:

(i) Effect of microwave treatment on BWF composites (BWF 0 compared to BWF 220):

Direct comparison between untreated and treated black wheat blends at equivalent substitution levels demonstrated that thermal processing significantly modified dough behavior. For BWF 220 at 10%, dough stability reached 10.33 min alongside a significant increase in setback torque (C5 = 3.354 Nm), indicating enhanced paste rigidity upon cooling. While initial torque values (C1 and C2) remained low, significant increases in C3 and C4 (*p* < 0.05) confirmed that microwave exposure promoted starch gelatinization and hot-paste enzymatic resistance. In untreated blends, BWF 0 (10%) exhibited a lower gluten index than BWF 0 (20%), suggesting that dough performance at higher substitution is controlled by starch–polyphenol filler interactions rather than functional gluten development. Correlation analysis (Figure S4) confirmed that in untreated BWF blends, dough stability is strongly correlated with protein-related parameters (C1/C2: r > 0.90, *p* < 0.01), whereas in microwave-treated BWF composites, stability was controlled by starch parameters (C3/C4 and C5: r > 0.85, *p* < 0.05).

(ii) Comparative functionality of microwave-treated flours (WWF 220 compared to BWF 220):

Comparing both thermally treated flours at identical 10% and 20% incorporation levels revealed different functional contributions to the carrier matrix. WWF 220 blends maintained marginally higher C1 and C2 values, indicating residual protein network retention. In contrast, BWF 220 composites demonstrated better retrogradation (higher C5) and paste stability. These rheological modifications directly reflect the combined impact of severe moisture reduction (<0.10%) and higher flour temperatures (151–152 °C; Table S2) reached at 220 s. At this level of exposure, rapid thermal dehydration leads to irreversible protein denaturation and rigid hyper-aggregation, validated by extensive disulfide cross-linking, which causes macroscopic gluten network collapse while simultaneously inducing starch crystallite rearrangement. These observations align with documented evidence that microwave irradiation compromises gluten integrity, yet simultaneously confers a degree of structural ordering to starch [5,23].

In summary, rather than characterizing individual flour cohesion, this rheological data highlights the interference and dilution dynamics of treated flours within composite dough systems. MW-treated WWF induces moderate gluten dilution while retaining minor mechanical resistance, whereas MW-treated BWF acts primarily as a functional starch-structuring component that enhances paste stability and retrogradation. These specific interactions support the practical feasibility of incorporating microwave-processed flours at 10–20% substitution in non-leavened products (pasta, biscuits, extruded snacks) to enhance binding and viscosity, rather than using them as conventional breadmaking formulations.

To the best of our knowledge, no studies to date have directly compared the rheological behavior of black wheat and whole wheat flours under microwave processing, highlighting the novelty and relevance of the present investigation.

### 3.2. Characterization of Starch

#### 3.2.1. FTIR-Derived Starch Short-Range Order

**Figure 2:**
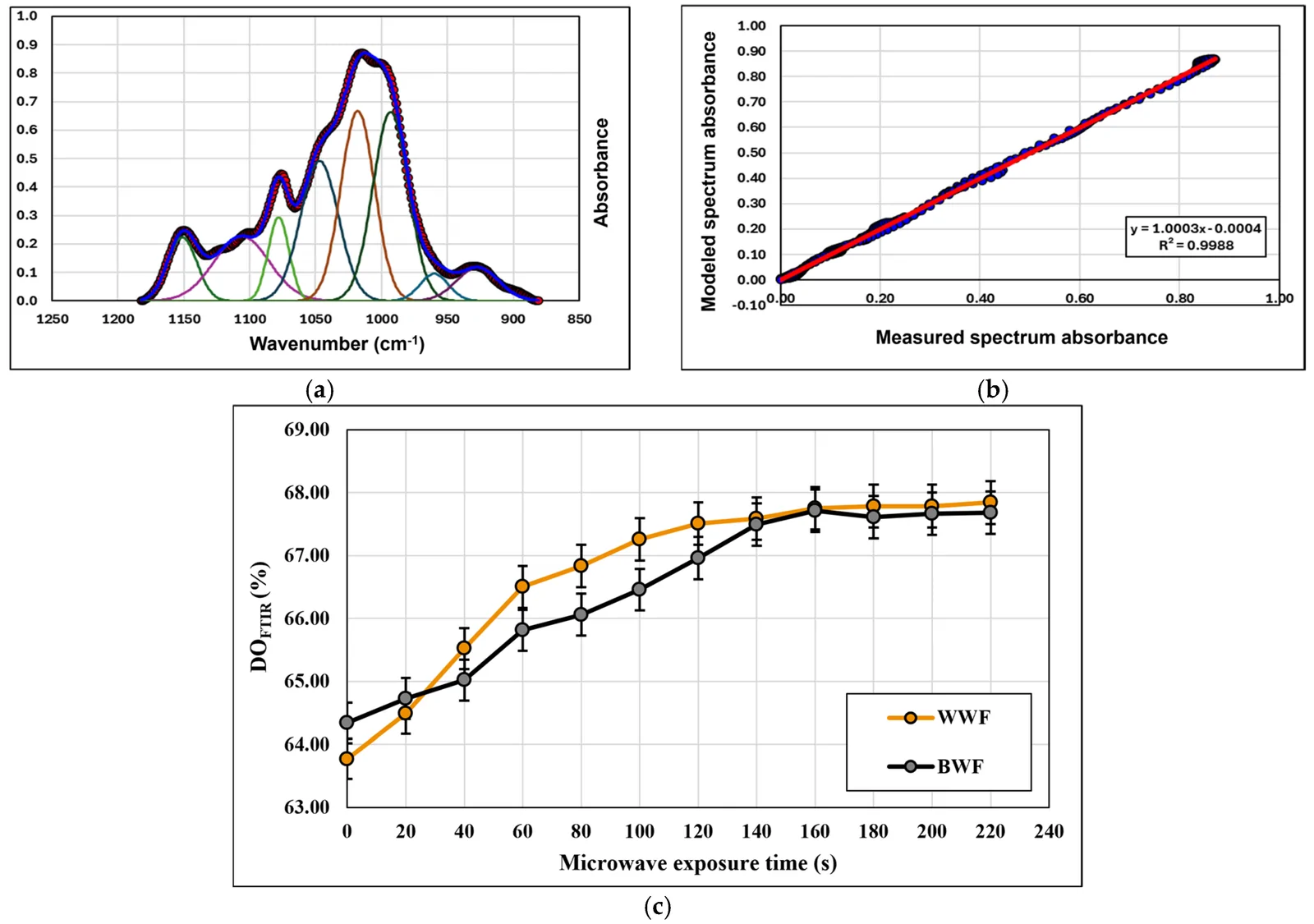
FTIR spectral deconvolution and short-range structural ordering: (**a**) determined Gaussian sub-bands within the 1200–850 cm^−1^ spectral domain; (**b**) regression validation between measured and modeled absorbance (R^2^ = 0.998); (**c**) dynamic changes in the degree of order (DO_FTIR_, % across microwave exposure times (0–220 s) for WWF and BWF. Values in (**c**) are presented as mean ± SD (*n* = 3).

The deconvoluted spectra for the starch domain are presented in Figure 2a,b. A detailed description of the FTIR deconvolution and interpretation procedures applied to starch and protein is provided in the Supplementary Material. Figure 2c illustrates the dependence of the FTIR-derived short-range order index (DO_FTIR_, %) in whole wheat flour (WWF) and black wheat flour (BWF) as a function of microwave exposure time, while Table 2 summarizes the ANOVA results.

Across both types of wheat flour, the structural evolution evolved in two different phases. During the initial exposure stage (0–100 s), a pronounced increase in DO_FTIR_ (%) was observed, due to thermal dehydration and the localized reassociation of amorphous starch structures into ordered double helices [28,29]. Beyond 120 s, DO_FTIR_ (%) stabilized, indicating the attainment of a conformational balance beyond which the proportion of ordered to amorphous domains remained relatively constant (*p* > 0.05).

ANOVA results (Table 2) confirmed these trends. Initial DO_FTIR_ (%) values were not significantly different between the two native flour samples (*p* > 0.05), with BWF displaying a baseline index of 64.34 ± 0.48% and WWF of 63.76 ± 0.16%. At maximum microwave exposure (200 s), both matrices exhibited significant structural ordering (*p* < 0.05), increasing by +6.38% for WWF and +5.18% for BWF. The higher increase observed in WWF is consistent with the findings of Zheng et al. [30] and could be attributed to its higher amylose content and lack of dense pigment encapsulation, which facilitates chain mobility and double-helical realignment. In BWF, the abundance of endogenous anthocyanins and free phenolics (Supplementary Material) [11,31] probably imposes steric hindrance and competitive hydrogen bonding, restricting the extent of polysaccharide reassociation [32,33].

These observations highlight the remarkable capacity of the starch matrix to undergo beneficial molecular rearrangements under microwave irradiation, leading to the thermodynamic consolidation and enhanced stability of its crystalline domains. Similar results were reported by Wang et al. [10], who found that microwave treatment followed by retrogradation promoted enhanced structural ordering of starch granules. Their study also revealed positive correlations between starch crystallinity and thermal/structural parameters such as transition onset temperature (T_0_), gelatinization enthalpy (ΔH), and the FTIR absorbance ratio 1047/1022 cm^−1^, supporting the concept that microwave energy facilitates the ordered restructuring of polysaccharide chains and the expansion of the crystalline phase.

#### 3.2.2. Atomic Force Microscopy

**Figure 3:**
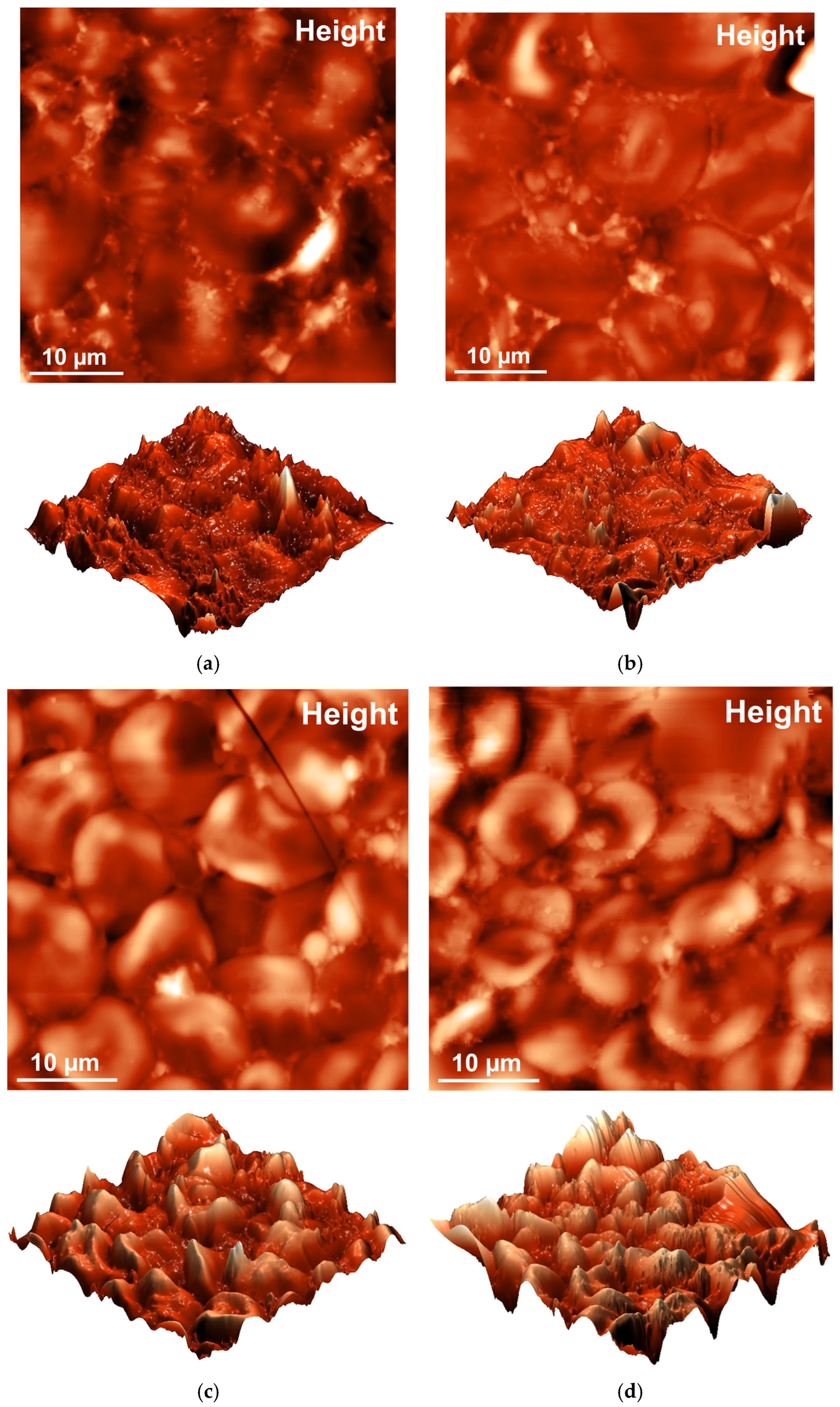
Topographic atomic force microscopy (AFM) micrographs depicting starch isolated from wheat samples before and after the microwave treatment: (**a**) native starch from WWF; (**b**) starch from WWF exposed to MW radiation for 120 s; (**c**) native starch from BWF; (**d**) starch from BWF exposed to MW radiation for 120 s.

The topographic images obtained by atomic force microscopy (AFM) of starch isolated from wheat samples prior to (t = 0 s) and following microwave treatment for 120 s are shown in Figure 3. The corresponding surface roughness parameters derived from AFM analysis are summarized in Table 2. AFM analysis of native starch isolated from whole wheat flour (WWF) (Figure 3a) revealed a relatively homogeneous surface, composed of well-defined granules predominantly lenticular to spherical in shape, with moderate dimensions, smooth edges, and a random yet uniform distribution. Surface roughness was relatively low (S_a_ = 68.70 nm, Table 2), and the texture remained consistent across the scanned area. Slight granule aggregation was observed, although without pronounced clustering. Occasional overlaps between adjacent particles suggested minor deformation or intergranular interactions, while smooth contours and faint radial striations indicated associations with internal lamellar structures or growth rings. These morphological attributes are consistent with previously reported structural models of starch organization, including the spherical block and concentric ring models [34,35].

Starch from untreated BWF exhibited higher variability in granule size and a stronger tendency toward aggregation than WWF, although individual granules maintained clear outlines and relatively smooth surfaces. After 120 s of microwave exposure, BWF showed more pronounced topographic changes than WWF, characterized by substantial increases in surface roughness and development of microfissures and depressions. These structural instabilities may be linked to the higher pigment and phenolic compound content in black wheat, which can interfere with starch matrix organization during thermal treatment [27].

During thermal treatment, the abundant hydroxyl groups of these phenolic compounds may competitively interact with water molecules for binding sites on the starch chains. They are hypothesized to establish non-covalent interactions with starch chains, primarily intermolecular hydrogen bonds and hydrophobic inclusion complexes with amylose [36]. This competitive complexation physically restricts the mobility of the starch polymers and prevents the optimal realignment of amylopectin double helices.

Consequently, the polyphenols could potentially act as structural disruptors, sterically hindering the ordered packing of the starch crystalline domains and leading to the observed structural instabilities and disordered matrix configuration during the microwave treatment [37]. Quantitative AFM analysis supported these observations. For WWF, the untreated control (WWF 0, Figure 3a) exhibited uniform surfaces with S_a_ = 68.70 nm and S_q_ = 99.50 nm. After 120 s of microwave treatment (Figure 3b), S_a_ and S_q_ increased significantly (*p* < 0.05) by 42% (S_a_ = 97.40 nm) and 30% (S_q_ = 129.30 nm), respectively. The effect was more pronounced in BWF (Figure 3c,d), where S_a_ increased by 39% (from 117.80 nm to 164.00 nm) and S_q_ by 41% (from 147.00 nm to 206.90 nm). These changes reflect enhanced fragmentation and disorder of the starch structure, attributed to thermal degradation and partial reorganization of amylose and amylopectin chains under microwave irradiation.

It is important to highlight that after a 220 s of MW exposure, AFM imaging was no longer feasible, as the surface roughness exceeded 1000 nm, resulting in signal instability and loss of focus. This suggests that prolonged microwave treatment induces severe morphological disruption beyond the resolution capabilities of AFM.

These findings are consistent with earlier studies [38,39] reporting that thermal and microwave treatments progressively increase starch surface roughness by expanding amorphous regions and reducing structural order. In the present study, the observed increase in surface roughness correlates with a higher order index detected by FTIR and with enhanced partial gelatinization (Section 3.1 and Section 3.2.1), highlighting the close interchange between microwave-induced thermal effects and starch morphological reorganization.

#### 3.2.3. X-Ray Diffraction

**Figure 4:**
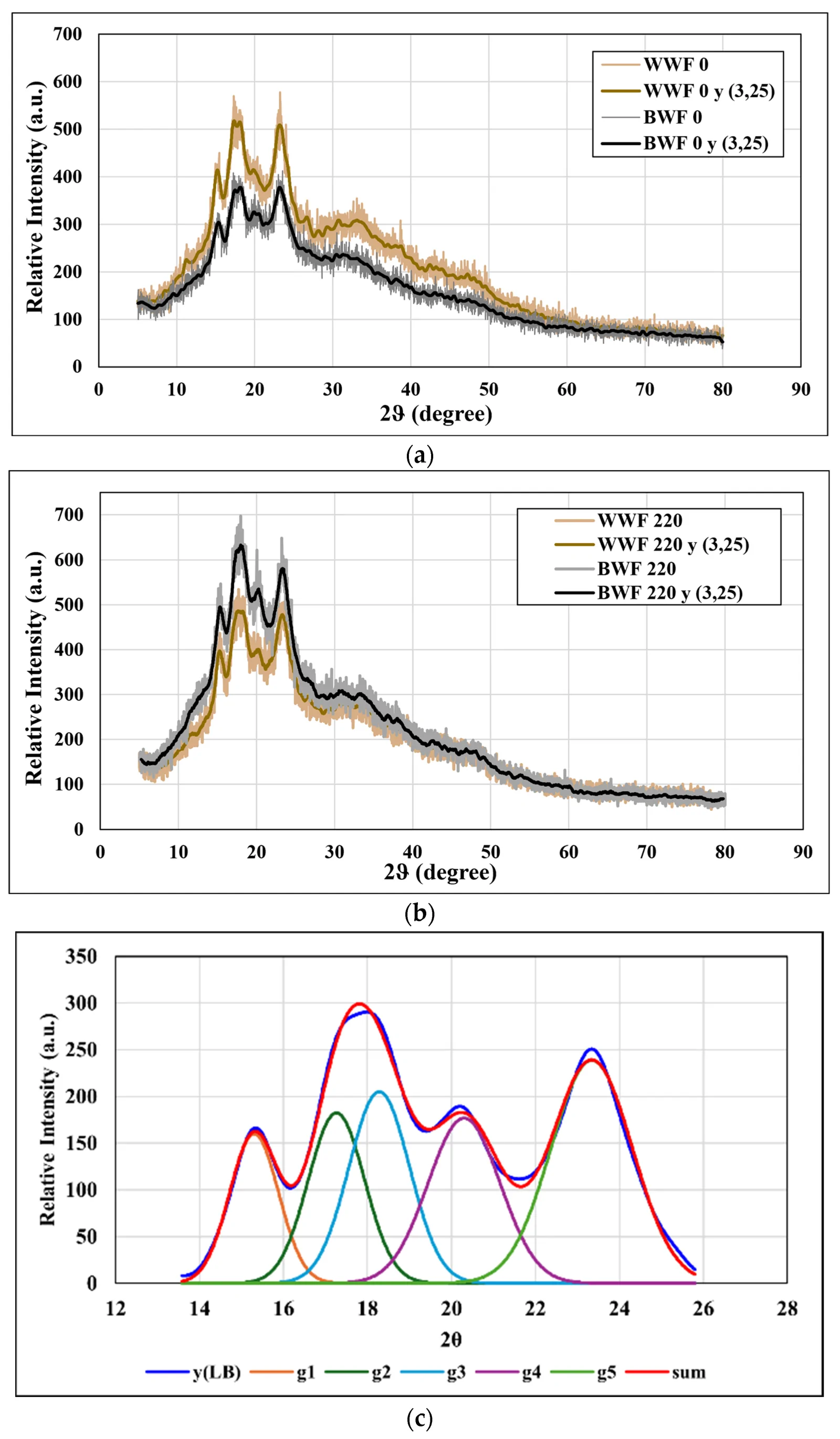
X-ray diffraction (XRD) patterns of whole wheat flour (WWF) and black wheat flour (BWF): (**a**) native untreated flours (WWF 0 and BWF 0); and (**b**) flours subjected to microwave thermal treatment for 220 s (WWF 220 and BWF 220). Thin, faint lines represent raw experimental diffraction profiles; bold continuous lines correspond to smoothed curves [y(3,25)] processed via a 25-point centered smoothing window; (**c**) Representative deconvolution of the crystalline XRD diffraction domain (2θ = 13–26°): y(LB) -baseline-corrected experimental diffraction pattern; g1–g5 represent individual resolved Gaussian crystalline reflections centered at 2θ ≈ 15.3° (g1), 17.2° (g2), 18.2° (g3), 20.2° (g4), and 23.4° (g5); sum—the cumulative fitted envelope (R^2^ > 0.99). Peaks g1, g2, g3, and g5 correspond to the typical A-type starch crystalline polymorph, whereas g4 reflects the *V_h_*-type single-helical amylose–lipid complex.

Lipids and amylose typically coexist in starch granules but recrystallize into Vh-type amylose–lipid complexes upon gelatinization [40]. X-ray diffraction (XRD) was used to identify starch polymorphs based on literature standards [4,41]: A-type starch exhibits characteristic peaks at approximately 2θ values of 15°, 17°, 18°, and 23°, while Vh-type polymorphs display distinctive peaks at 7.50°, 13.00°, and 19.50°. To quantify these phases in WWF and BWF, the diffractograms (Figure 4a,b) were deconvoluted (Figure 4c and Figure S2). Table 2 presents the total crystallinity and the distribution of A- and Vh-type fractions.

Complementing FTIR short-range order data, XRD evaluated the long-range crystallinity and showed similar trends in both flour samples. After 220 s of MW exposure, total crystallinity considerably increased (*p* < 0.05), from 20.49% to 26.59% (+29.77%) in WWF and from 26.78% to 31.45% (+17.53%) in BWF. This intensification was primarily due to an increase in A-type crystallinity (+26.16% in WWF, +14.29% in BWF), as well as a pronounced enhancement of Vh-type structures (+47.73% in WWF, +32.20% in BWF), which could be explained by amylose retrogradation and the formation of amylose–lipid complexes [4]. This structural rearrangement was determined by the combined effect of progressive moisture evaporation (<0.10% beyond 160 s) and microwave-induced thermal accumulation (reaching 151–152 °C at 200 s; Table S2), which promote biopolymer mobility and subsequent crystallite reorganization. These findings are consistent with the FTIR results (Section 3.2.1, Figure 2, Table 2), which also revealed increases in DO_FTIR_ (%), from 63.76% to 67.84% (+6.38%, *p* < 0.05) in WWF and from 64.34% to 67.68% (+5.18%, *p* < 0.05) in BWF. Strong correlations (Figure S4) were observed between FTIR and XRD data (r = 0.91, *p* < 0.01 for WWF and r = 0.88, *p* < 0.01 for BWF) confirming the reliability of FTIR as a complementary tool for crystallinity assessment.

Correlating the structural changes described above with Mixolab rheological parameters (Table 1, Figure S4) reveals a dependent relationship between starch crystallinity and dough functionality. Increased crystallinity (+29.77% in WWF; +17.53% in BWF) was associated with reductions in C1 and C2, reflected by strong negative correlations (r = −0.95 and r = −0.94, *p* < 0.01), indicating lower gluten development and reduced protein thermal stability, particularly in microwave-exposed samples. In contrast, increases in C3 and C4 values confirmed a more efficient starch gelatinization and enhanced enzymatic resistance (r = 0.84, *p* < 0.05), results that can be directly attributed to the reorganization of crystalline domains observed in XRD and FTIR. Moreover, higher C5 values in MW-treated samples (for example, WWF 220 s: 2.998 Nm compared to an initial 2.516 Nm), which positively correlated with total crystallinity (r = 0.98, *p* < 0.01), demonstrated intensified starch retrogradation during cooling, in agreement with the observed rise in Vh-type crystallinity (+47.73% in WWF, +32.20% in BWF). These results suggest that accelerated starch recrystallization, as evidenced by XRD, results in improved water retention and the formation of more stable retrograded structures, which is linked to the increased rheological parameters of viscosity and dough stability.

Unlike WWF, BWF exhibited remarkable stability of crystalline fractions after microwave exposure. Thermal and moisture monitoring (Table S2) confirmed that moisture levels equalized as early as 40 s (~6.8%) and final temperatures converged (151–152 °C), validating that this relative stability was not an artifact of different thermal or dehydration profiles. It is likely attributed to its specific anthocyanins and phenolic compounds, which stabilize starch chains via non-covalent interactions, thereby protecting both A and Vh-type domains [42]. These results align with the findings of Qiu et al. [43] regarding the thermal reinforcement of starch crystallinity in cereal matrices. XRD, FTIR, and rheological data confirm that microwave treatment significantly reorganizes starch structure, specifically enhancing Vh-type crystallinity. MW processing offers a viable method to tailor the functional and nutritional potential of colored cereal matrices by modulating their crystalline architecture.

#### 3.2.4. Scanning Electron Microscopy (SEM)

Table 3 presents scanning electron microscopy (SEM) micrographs illustrating the morphology of starch granules isolated from WWF and BWF, comparatively analyzed before and after microwave exposure. SEM provides complementary evidence to AFM and FTIR/XRD analyses, enabling the visualization of surface features and structural modifications induced by thermal treatment at starch-granule level.

#### 3.2.5. In Vitro Starch Digestion Kinetics and Nutritional Fractions

**Figure 5:**
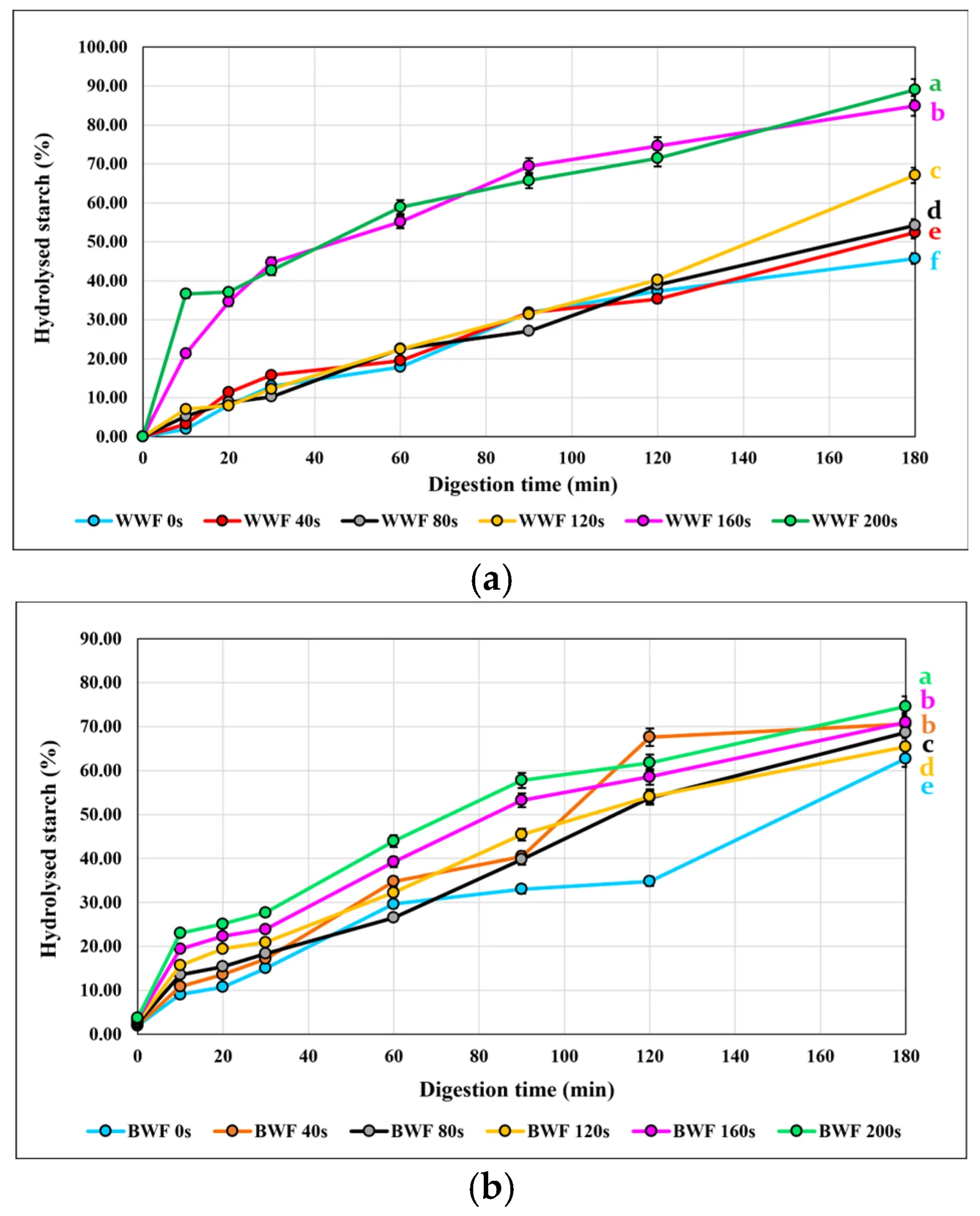
Dynamic in vitro starch hydrolysis curves over 180 min for: (**a**)—whole wheat flour (WWF); and (**b**)—black wheat flour (BWF) subjected to microwave thermal treatment for 0–200 s. Data points represent the mean ± standard deviation (*n* = 3). Different lowercase letters (a–f) at the final digestion time (180 min) indicate statistically significant differences among microwave exposure durations within the same flour type (*p* < 0.05, one-way ANOVA followed by Tukey’s HSD post hoc test).

The microstructural alterations revealed by SEM, particularly the progressive granular degradation, surface pitting, and partial fragmentation, suggested significant changes in the accessibility of the starch matrix to solvent penetration. To quantitatively verify whether these morphological disruptions resulted into altered digestive behavior, the dynamic in vitro hydrolysis kinetics and nutritional starch fractions (RDS, SDS, and RS) were evaluated (Figure 5a,b, Table 4).

The in vitro digestion profiles (Figure 5a,b; curve fittings in Supplementary Figure S5) and kinetic parameters (Table 4) demonstrated treatment-dependent starch digestibilities (R^2^ = 0.95–0.99).

Untreated samples (0 s) exhibited high resistant starch fractions (62.67% in WWF and 65.23% in BWF) and restricted hydrolysis rates (k ≈ 0.0055–0.0061 min^−1^), reflecting intact granular envelopes. Moderate microwave heating (40–120 s) revealed different matrix behaviors: WWF retained its structural resistance (RS = 59.76–64.66%; RDS < 11.5%; k ≈ 0.0048–0.0065 min^−1^), indicating that mild dehydration did not disrupt the dense gluten–pericarp matrix. On the other hand, BWF underwent a substantial redistribution toward slowly digestible starch (SDS increasing from 24.04% to 52.22% at 80 s, *p* < 0.05) alongside a gradual increase in hydrolysis rate (k = 0.0068–0.0113 min^−1^). This SDS accumulation could be attributed to partial starch swelling coupled with non-covalent starch–polyphenol entrapment by endogenous anthocyanins.

Extending exposure to 160–200 s caused a pronounced kinetic shift due to structural collapse and incipient dry pyrolysis. In WWF, k increased four-fold at 160 s (0.0242 min^−1^) and peaked at 200 s (0.0441 min^−1^), accompanied by high RDS content (37.08%). Under severe thermal conditions (>150 °C, moisture < 0.10%), the sharp increase in DNS reducing sugars reflects extensive thermal dextrinization and pyrolytic chain scission rather than standard enzymatic hydrolysis only. Furthermore, the apparent decrease in *C*_∞_ for WWF at 200 s (67.03% compared to 80.76% at 160 s) reflects advanced charring, which locked a fraction of the matrix into insoluble, enzymatically inaccessible carbonaceous aggregates [34]. In contrast, BWF underwent a more tempered transition (k = 0.0142–0.0171 min^−1^; RDS = 22.30–25.08%) demonstrating enhanced thermal buffering conferred by its pigmented bran.

Since HI and eGI were calculated relative to native flours (0 s = 100%), severely treated samples (160–200 s) yielded values exceeding 200% for HI and 150% for eGI. These results confirm that the combined effects of envelope rupture, dextrinization, and thermal degradation accelerated early enzymatic glucose release more than two-fold relative to the dense native flour matrix.

### 3.3. Characterization of Wheat Protein

#### 3.3.1. FTIR Analysis of the Protein Profile

**Figure 6:**
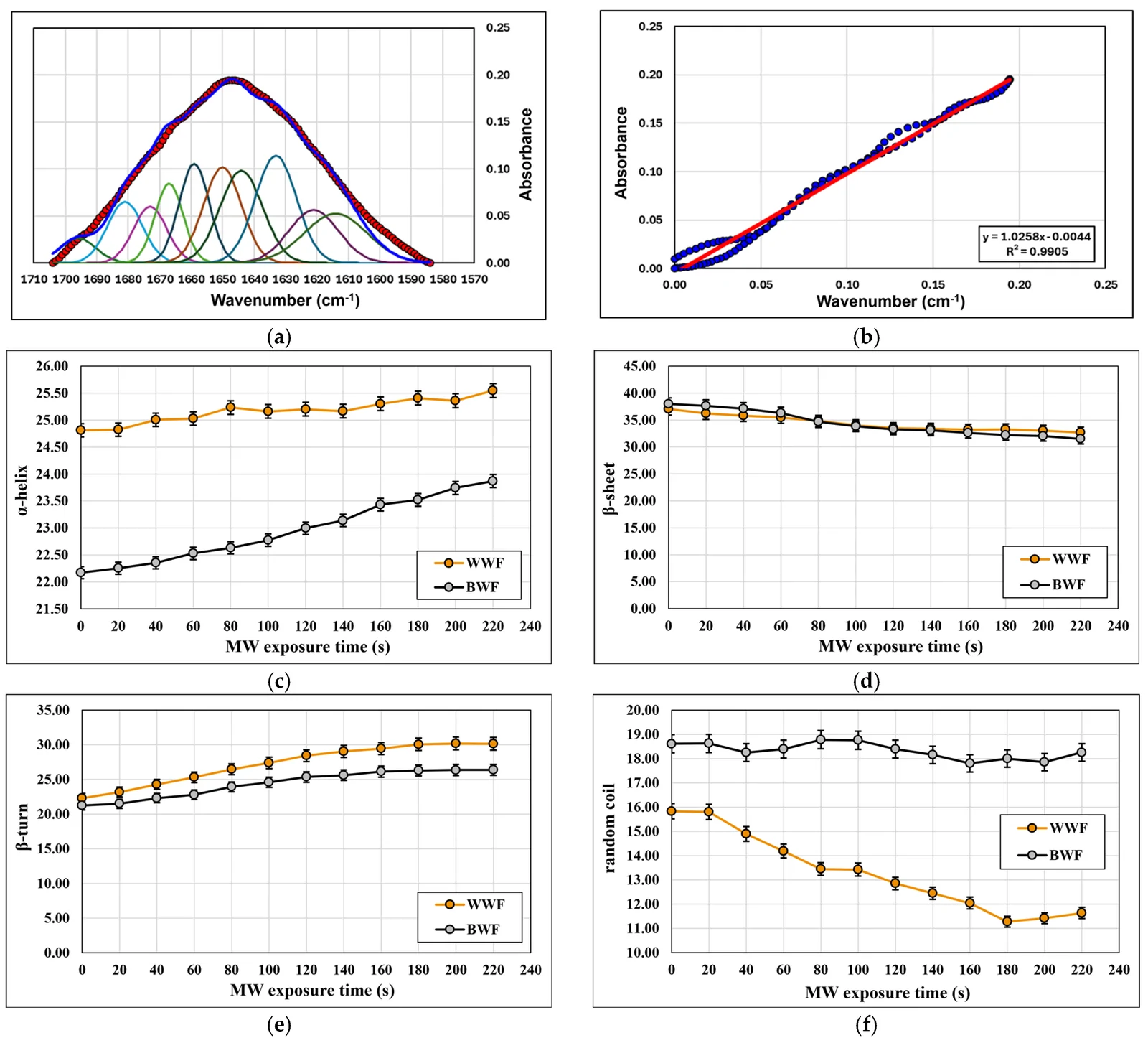
FTIR spectral deconvolution of the protein domain: (**a**) Determined Gaussian sub-bands in the amide I region (1710–1580 cm^−1^); (**b**) linear regression between experimental and modeled absorbance validating the spectral deconvolution accuracy (R^2^ = 0.9905); (**c**–**f**) changes in secondary protein structure conformations under microwave exposure.

Deconvoluted FTIR profiles within the protein amide I region are displayed in Figure 6a,b, while Figure 6c–f illustrate the time-dependent restructuring of protein secondary conformations, tracking changes in the relative proportions of α-helices, β-sheets, β-turns, and random coils throughout microwave exposure.

Two dominant trends, highly relevant for interpreting the structural response of the analyzed samples, can be distinguished: (i) The analysis revealed either moderate increases or relative stability in the proportions of α-helix and β-turn structures during microwave treatment, while β-sheet and random coil conformations showed progressive decreases (*p* < 0.05) with increasing of exposure time. (ii) At baseline (t = 0 s), different structural differences were observed. WWF displayed the highest proportion of α-helix (24.82% compared to 22.17% in black wheat flour, *p* < 0.05), indicative of more ordered and stable conformations. In contrast, BWF was enriched in β-sheet (37.97% compared to 37.04% in WWF, *p* > 0.05) and random coil structures (18.61% compared to 15.83% in WWF, *p* < 0.05), suggesting a more flexible and structurally heterogeneous protein architecture [44,45]. Slightly higher β-turn content was also noticed for WWF (22.32% compared to 21.24%, *p* < 0.05). These initial differences reflect characteristic compositional and structural features that likely influence the subsequent microwave response.

Prolonged MW exposure induced statistically significant conformational changes in both types of flour (Table 5). In WWF, α-helix content increased from 24.82% to 25.55% (+2.96%, *p* < 0.05), coupled by a significant β-turns increase from 22.32% to 30.14% (+35.05%, *p* < 0.05). In contrast, a decrease was observed for β-sheets by 11.77% (from 37.04% to 32.68%, *p* < 0.05) and for random coils by 26.50% (from 15.83% to 11.64%, *p* < 0.05). This transition reflects conformational destabilization induced by thermal denaturation, characterized by the disruption of native, ordered β-sheets and polypeptide unfolding into transitional loop conformations. In the case of BWF, the structural changes were moderate, consistent with better conformational resistance under microwave treatment. Specifically, α-helix increased by 7.67% (from 22.17% to 23.87%, *p* < 0.05) and β-turn increased by 24.15% (from 21.24% to 26.38%, *p* < 0.05), while β-sheet decreased by 17.06% (from 37.97% to 31.49%, *p* < 0.05). Random coil displayed statistically insignificant changes (−1.91%, *p* > 0.05). This relative structural stability of BWF is likely mediated by non-covalent interactions with endogenous phenolics and anthocyanins, which exert a steric shielding effect that restricts thermal unfolding along the polypeptide backbone [46].

The cumulative structural index (the sum of α-helix + β-sheet + β-turn) increased in WWF from 84.18% to 88.37%. Importantly, from a polymer physics perspective, this molecular-level ordering cannot be interpreted as an improvement in baking performance. While the transition from disordered structures toward ordered conformations reflects local thermodynamic stabilization, excessive rigidification, determined by intensive thermal dehydration and disulfide cross-linking, induces irreversible thermal denaturation and hyper-aggregation. This structural over-curing eliminates the conformational mobility and elasticity essential for gluten hydration and network extension, directly explaining the macroscopic dough collapse and the complete inability of pure treated flour (WWF 220, 100%) to develop a workable dough in the Mixolab analysis. Meanwhile, the secondary structure of BWF remained unchanged (81.38–81.74%), demonstrating significant conformational restriction rather than active functional network formation. In BWF, structural locking through endogenous phenolic and anthocyanin complexes prevented excessive thermal unfolding yet completely restricted the dynamic viscoelastic rearrangement required for dough development. These molecular findings directly underpin the rheological results, demonstrating that microwave-induced structural ordering represents network rigidity and thermal inactivation rather than enhanced dough elasticity.

These results demonstrate that WWF proteins undergo pronounced thermal restructuring toward rigidified conformations under microwave exposure [9,47], whereas BWF proteins remain conformationally locked. These molecular differences align with the composite rheological trends, where WWF suffered extensive thermal network disruption, while BWF functioned primarily through starch-dominated filler mechanics rather than active gluten development.

#### 3.3.2. Surface Hydrophobicity

**Figure 7:**
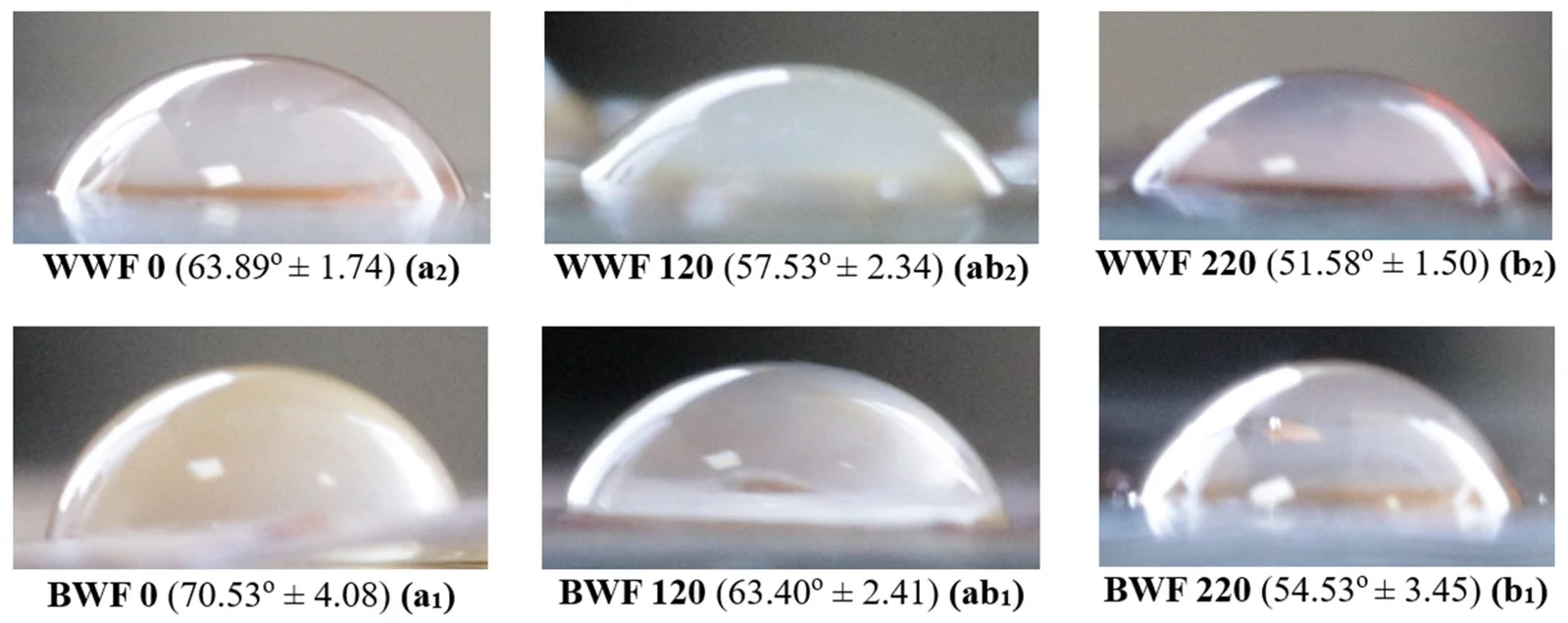
Surface hydrophobicity of protein isolates from two types of wheat flour subjected to microwave treatment, where: WWF 0—whole wheat flour before MW exposure; BWF 0—black wheat flour before MW exposure; WWF 120—whole wheat flour treated with microwave irradiation for 120 s; BWF 120—black wheat flour treated with microwave irradiation for 120 s; WWF 220—whole wheat flour treated with microwave irradiation for 220 s; BWF 220—black wheat flour treated with microwave irradiation for 220 s. Results are expressed as mean values ± standard deviations (*n* ≥ 3). Different letters (a, b) within the same row indicate statistically significant differences (*p* < 0.05) between means (Tukey’s test). Different numbers (1, 2) within the same column indicate statistically significant differences (*p* < 0.05) between means (Tukey’s test).

Microwave exposure induced significant changes in the surface properties of the protein isolates, mostly reflected by a progressive reduction in hydrophobicity. This trend, of conformational reorganization during exposure, was confirmed by the decrease in contact angle values relative to native flour samples (Figure 7). In WWF, the contact angle decreased significantly from 63.89° ± 1.74° at t = 0 s to 51.58° ± 1.50° after 220 s of microwave exposure, corresponding to a reduction of approximately 19.2% (*p* < 0.05). Similarly, BWF exhibited a decrease from 70.53° ± 4.08° to 54.53° ± 3.45°, equivalent to a 22.70% reduction (*p* < 0.05). Collectively, these results demonstrate that microwave irradiation reduces protein surface hydrophobicity, most likely due to conformational changes also evidenced by FTIR analysis (Section 3.3.1), which revealed increases in α-helix and β-turn content and decreases in β-sheet and random coil fractions.

Protein hydrophobicity plays a crucial role in determining stability, conformation, and digestibility of wheat proteins [48]. The observed decrease may be attributed to aggregation phenomena, where intense hydrophobic interactions during heating promote a “hydrophobic collapse”, sending non-polar groups into the protein interior and thereby lowering surface hydrophobicity [49]. At high temperatures (>80 °C), microwave energy may additionally disrupt ionic and non-covalent bonds, transiently exposing hydrophobic residues [10]. Subsequent conformational rearrangements, including the reorientation of polar residues toward the molecular surface, provide a possible explanation for the net reduction in hydrophobicity observed in this study.

Compared to WWF, BWF initially exhibited higher surface hydrophobicity (H_0_). The planar aromatic rings of phenolic compounds in BWF interact with the gluten network, inducing partial protein unfolding and exposing previously buried hydrophobic groups [50]. However, microwave exposure significantly decreased contact angles in both flours, reflecting a shift toward overall surface hydrophilicity. While microwave irradiation initially causes denaturation and transiently exposes hydrophobic groups [45], subsequent thermal aggregation rapidly re-buries these regions. This network reorganization forces polar amino-acid residues outward, promoting extensive hydrogen bonding with water molecules [20].

From a technological perspective, the reduction in protein surface hydrophobicity may have beneficial implications for food processing. It could improve solubility, foaming capacity, and emulsifying properties, functional parameters that are essential for the development and quality of certain cereal-based products.

#### 3.3.3. Free -SH, Total -SH and -SS Content in Wheat Protein Isolate

Free thiol groups (-SH), located in cysteine residues of polypeptide chains, are highly reactive and actively participate in oxidation reactions and thiol–disulfide (-SH/-S-S-) exchange. Through oxidation, free thiols can form disulfide bonds (-S-S-), covalent linkages essential for the stabilization of the tertiary and quaternary protein structures and, consequently, for strengthening the gluten network in dough [51,52]. In parallel, bound thiols, integrated within the protein backbone (particularly in glutenins and gliadins), also take part in -SH/-S-S- interchange reactions, thereby influencing the reorganization and functional performance of the protein structure [53].

Comparative analysis of the samples (Table 5) revealed differences from the native state (t = 0 s). The concentration of free sulfhydryl groups (-SH) was significantly higher in BWF protein isolate (4.78 ± 0.06 µmol/g) compared to WWF isolate (2.44 ± 0.01 µmol/g, *p* < 0.05). Total -SH content followed a similar trend, being more than three times higher in BWF (14.39 ± 0.08 µmol/g) relative to WWF (4.48 ± 0.03 µmol/g). Furthermore, the initial values of -S-S- were significantly (*p* < 0.05) higher in BWF (4.80 ± 0.05 µmol/g) compared to WWF (1.02 ± 0.02 µmol/g). This baseline profile suggests that BWF proteins naturally possess a more complex structure characterized by a higher degree of cross-linking, alongside a larger reserve of reactive -SH groups compared to WWF.

Microwave exposure induced structural modifications between the two matrices. In the case of WWF, thermal processing predominantly promoted oxidative cross-linking: free -SH content significantly decreased (−64.75% at 120 s and −59.01% at 220 s), while disulfide bonds (-SS) exhibited a pronounced increase of +419.60% (5.30 ± 0.04 μmol/g) after 220 s of exposure. Because disulfide content is algebraically derived from the difference between total and free -SH ([Total -SH -Free -SH]/2), this substantial percentage increase is mathematically amplified by the low baseline disulfide value (1.02 μmol/g) of native WWF. Alongside, the measured total -SH in WWF increased by +159.15% at 220 s. In a closed system where absolute cysteine abundance is conserved, this phenomenon does not represent de novo synthesis of sulfur-containing groups; rather, it reflects an increase in chemical accessibility [54]. The combined impact of high temperatures (151–152 °C) and severe moisture loss (<0.10%; Table S2) induces extensive thermal unfolding and disruption of compact, sterically hindered hydrophobic glutenin aggregates, thereby exposing previously buried cysteine residues to the denaturing buffer and Ellman’s reagent [55]. Consequently, the thermal conversion of newly exposed free -SH into inter-chain -S-S- bridges indicates substantial rigidification and heat-induced aggregation of the WWF protein network [20,56].

In the case of BWF, the evolution was different. Under moderate exposure (120 s), a decrease in all parameters was observed (−56.90% for free -SH, −12.29% for -SS), pointing to initial protein aggregation and steric masking of reactive sites. Extending exposure to 220 s, however, resulted in a sharp increase in free -SH (+75.52%, reaching 8.39 µmol/g) and a moderate recovery of -SS bonds (+13.75%). This simultaneous increase in both free and total -SH groups suggests advanced protein unfolding that exposes substantial quantities of native -SH groups. Unlike in WWF, where free -SH groups were rapidly converted into disulfide cross-links, the abundant endogenous polyphenols and anthocyanins in BWF (Table S1) likely establish a reducing, antioxidant microenvironment (Supplementary Material) [57] that hampers the conventional heat-induced oxidative pairing of -SH into -SS bridges. While secondary reactions such as non-disulfide oxidation pathways or quinone–thiol covalent adduct formation have been hypothesized in literature under high-temperature roasting [48,58], they were not directly measured by the spectrophotometric Ellman assay employed here. Nonetheless, the persistence of high free -SH groups and limited net disulfide cross-linking explain the macroscopic behavior of BWF: the inability to form a cohesive, elastomeric gluten network under Mixolab shear is directly attributable to this lack of rigid intermolecular -SS cross-linking, consistent with previous reports on pigmented wheats [11,59].

These results correlate closely with FTIR and rheological data, confirming a less organized protein network characterized by reduced β-sheets and increased disordered fractions. Despite a slight increase in -SS bonds, the massive increase in free -SH groups indicates conformational instability. Pearson analysis confirmed this: -S-S- correlated positively with stability (r = 0.65), while -SH negatively impacted elasticity (r = −0.70, *p* < 0.01). Additionally, the altered surface properties reflected modified polarity due to functional group exposure. While these changes compromise conventional gluten strength in high-volume breadmaking, they represent advantageous functional traits for applications requiring extensible, low-elasticity matrices (such as biscuits, wafers, or flatbreads) (Supplementary Material) [60]. In such applications, reduced elasticity limits dough shrinkage (snap-back) while promoting spread and baked tenderness, effectively positioning BWF as an intrinsic, elasticity-attenuating ingredient. From a nutritional perspective, this conformational remodeling, shown by preserved α-helices and decreased dense β-sheets, may improve enzymatic accessibility and in vitro protein digestibility in MW-treated black wheat.

#### 3.3.4. Interactions Between Starch and Proteins, Caused by MW Exposure

Microwave dielectric heating induced coupled macromolecular reorganizations between the starch and proteins in both flours, controlled by internal vapor pressure generation and rapid thermal dissipation. Volumetric heating facilitated partial amylose leaching and granular surface disruption, promoting the formation of V-type amylose–lipid complexes (XRD/FTIR) alongside non-covalent associations between starch chains and unfolding polypeptide segments. The protein fraction underwent progressive thermal denaturation rather than constructive structural ordering: native β-sheets decreased (from 37.04% to 32.68% in WWF and 37.97% to 31.49% in BWF), and β-turn structures increased (WWF: 22.32% to 30.14%; BWF: 21.24% to 26.37%).

Rather than reinforcing dough cohesion, this structural shift caused severe network stiffening in WWF through extensive disulfide polymerization (+419.6%). On the other hand, BWF preserved flexible random coils (~18.3%) and free thiols (+75.5%) through steric shielding by endogenous anthocyanins and phenolics, sustaining its role as an elasticity-attenuating component. Simultaneously, granular thermal fissuring and elevated nanoscale roughness facilitated enzymatic access, accelerating in vitro amylolysis and increasing rapidly digestible starch (RDS) and eGI. Similar effects have been reported by Zhang et al. [61], who observed compact aggregates through starch–protein interactions. Also, Zhang et al. [62] showed that microwaves promote starch–polyphenol complexation, altering crystallinity and digestibility, confirming that dielectric heating governs techno-functionality by matrix-specific degradation pathways.

#### 3.3.5. PCA Analysis

**Figure 8:**
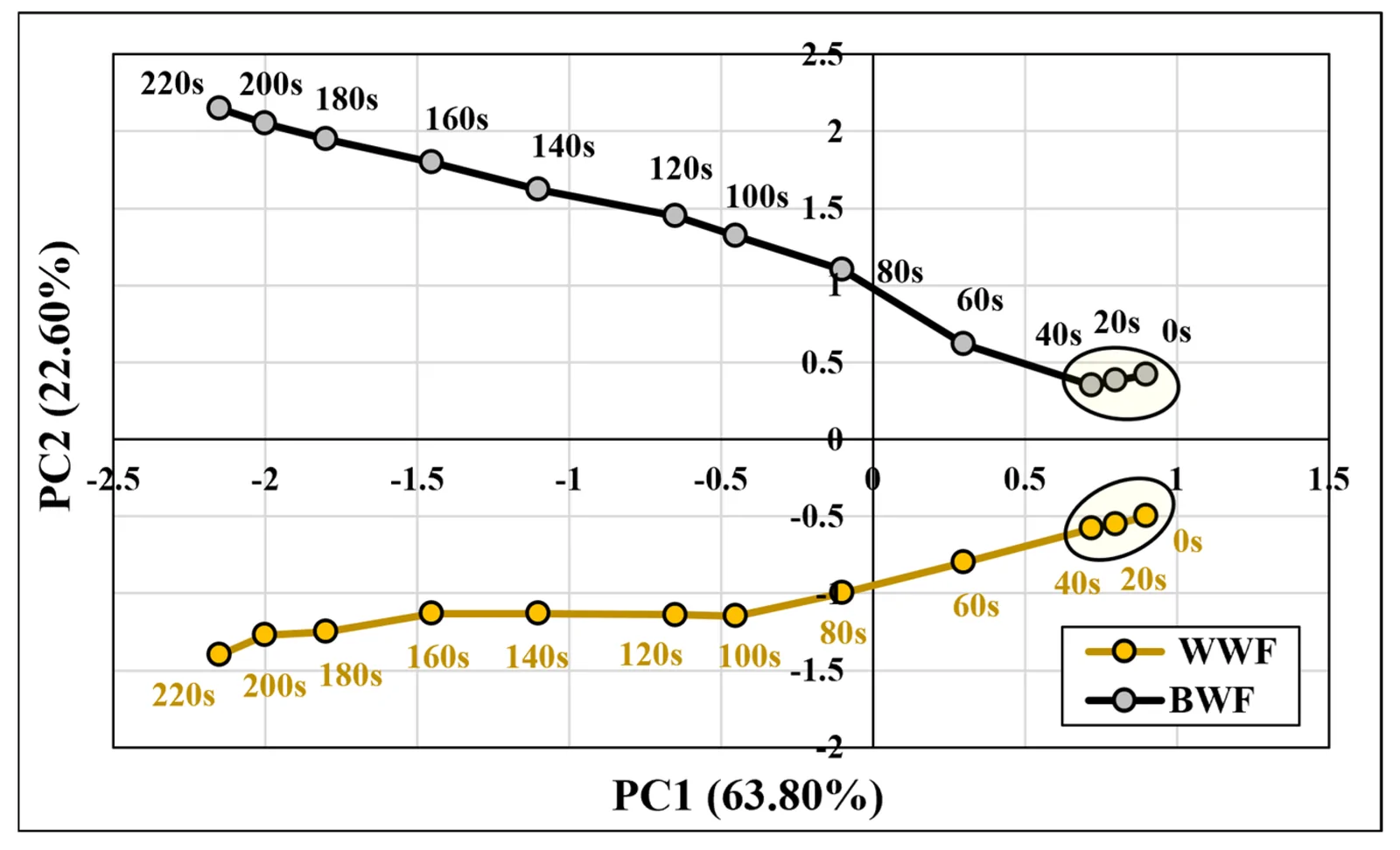
PCA score plot of macromolecular conformations and starch digestion dynamics in WWF and BWF across microwave exposure times (0–220 s).

Principal component analysis (PCA) accounted for 86.40% of the total cumulative variance across PC1 (63.80%) and PC2 (22.60%), clearly separating thermal processing severity from matrix-specific biopolymer mechanics (Figure 8).

PC1 (Processing severity and digestion) delineated the progressive thermal breakdown and starch digestion trajectory. Native flours (0 s) clustered in the positive PC1 domain, characterized by native β-sheets and high resistant starch fractions (RS > 62%). Microwave exposure drove samples toward negative PC1 coordinates due to granular disruption and partial gelatinization, which facilitated amylolytic accessibility and expanded the rapidly digestible starch fraction (RDS reaching 37.1% in WWF and 25.1% in BWF at 200 s), accelerating digestion rate (k) and expected glycemic index (eGI).

PC2 (Protein conformational difference): BWF migrated exclusively into the positive PC2 domain (+2.15 at 220 s), supported by preserved flexible random coils (17.8–18.6%) and slowly digestible starch (SDS), where polyphenol–anthocyanin complexes sterically hindered rigid disulfide cross-linking. In contrast, WWF shifted into the negative PC2 space (−1.40 at 220 s) due to severe thermal hyper-aggregation and covalent rigidification (β-turns increasing from 22.3% to 30.1%), eliminating the conformational flexibility required for gluten network cohesion.

Overall, while microwave radiation uniformly accelerates starch amylolysis along PC1, it separates protein structural dynamics along PC2. This distinction validates BWF as a component for non-leavened food products and confirms that severely treated WWF must be restricted to composite flour formulations.

## 4. Conclusions

This study demonstrates that microwave treatment induces significant structural, conformational, and digestive reorganizations within the biopolymeric matrix of whole-grain wheat flours, distinguishing the behaviors of whole wheat flour (WWF) and black wheat flour (BWF). The integration of XRD and FTIR analyses revealed the formation of Vh-type polymorphs and a transition of proteins towards ordered α-helical structures. Morphological disruption and granular fissuring observed in SEM directly regulated in vitro digestion behavior, leading to improved enzymatic accessibility, an increased rapidly digestible starch (RDS) fraction, and a high estimated glycemic index (eGI). Comparatively, BWF exhibited higher structural resistance under thermal stress, supported by a tempered digestion rate (k), prolonged preservation of slowly digestible starch (SDS), and higher residual resistant starch (RS). Rather than attributing this stability completely to grain pigmentation, this enhanced integrity is plausibly given by the flour’s phytochemical composition, specifically its diverse protein and fiber fractions, coupled with protective non-covalent associations involving its rich phenolic and anthocyanin content. Technologically, the compromised granular envelope and hydration capacity make microwave-treated flours particularly well suited for formulating firm-textured, rapid-hydration products such as biscuits, extruded snacks and flatbreads, where a reorganized protein network and tailored pasting properties offer clear quality advantages. In these applications, the increased hydration capacity, modified pasting profiles, and suppressed disulfide cross-linking in BWF provide distinct functional advantages by reducing dough shrinkage (snap-back) and promoting baked tenderness. However, as the proposed stabilization mechanism involving starch–protein–phenolic complexes is currently inferred from rheological and spectroscopic correlations, future research requires direct molecular isolation to definitively characterize these supramolecular assemblies. Additionally, data on long-term storage stability, bioactive retention, and industrial scalability are required to validate commercial viability.

## Figures and Tables

**Table 1:** Rheological characteristics of WWF and BWF before and after microwave exposure.

Sample	Hydration Capacity (%)	Dough Stability (min)	C1 (Nm)	C2 (Nm)	C3 (Nm)	C4 (Nm)	C5 (Nm)	C4/C3 (Nm)
WWF 0 (100%)	60	10.40	1.628	0.816	2.111	1.658	2.516	0.79
WWF 220 (10%)	60	9.30	1.177	0.607	1.700	1.846	2.998	1.08
WWF 220 (20%)	60	8.97	1.296	0.675	1.978	1.904	2.979	0.96
BWF 0 (10%)	60	9.37	1.144	0.573	1.708	1.867	2.881	1.09
BWF 0 (20%)	60	8.53	1.206	0.530	1.767	1.857	2.821	1.05
BWF220 (10%)	60	10.33	1.046	0.540	2.029	1.847	3.354	0.91
BWF 220 (20%)	60	9.98	1.073	0.511	1.992	1.926	2.968	0.96

C1—dough development capacity and resistance to mixing; C2—protein stability and susceptibility to thermal weakening; C3—starch gelatinization capacity; C4—hot-paste stability and cooking resistance under continuous shear; C5—starch retrogradation during cooling; C4/C3—baking stability; WWF 0 (100%)—whole wheat flour not subjected to microwave treatment; WWF 220 (10%)—mix of whole wheat flour (10%) treated with microwave irradiation for 220 s and white wheat flour (90%); WWF 220 (20%)—mix of whole wheat flour (20%) treated with microwave irradiation for 220 s and white wheat flour (80%); BWF 0 (10%)—mix of black wheat flour (10%) not subjected to microwave treatment and white wheat flour (90%); BWF 0 (20%)—mix of black wheat flour (20%) not subjected to microwave treatment and white wheat flour (80%); BWF 220 (10%)—mix of black wheat flour (10%) treated with microwave irradiation for 220 s and white wheat flour (90%); BWF 220 (20%)—blend of black wheat flour (20%) treated with microwave irradiation for 220 s and white wheat flour (80%).

**Table 2:** Starch crystallinity, surface roughness, and protein structure of wheat flour samples.

FTIR Short-Range Order Index (1047/1020 cm^−1^)						
Parameter		WWF			BWF	
Before exposure (t = 0 s)		63.76 ± 0.16 (a_2_)			64.34 ± 0.48 (a_2_)	
After exposure (t = 220 s)		67.84 ± 0.26 (a_1_)			67.68 ± 0.21 (a_1_)	
Variation (%)		+6.38			+5.18	
Surface roughness of starch granules isolated from whole and black wheat flour, in native state and after MW treatment, as determined by AFM						
Sample	Non-exposed sample (t = 0 s)			MW exposed sample (t = 120 s)		
	Average roughness S_a_ (nm)		Medium square root S_q_ (nm)	Average roughness S_a_ (nm)		Medium square root S_q_ (nm)
WWF	68.70		99.50	97.40		129.30
BWF	117.80		147.00	164.00		206.90
Crystallinity degree obtained from XRD histogram analysis (%)						
Total degree of crystallinity (%)						
Samples		WWF			BWF	
Before exposure (t = 0 s)		20.49 ± 1.00 (b_2_)			26.78 ± 1.66 (a_2_)	
After exposure (t = 220 s)		26.59 ± 1.35 (b_1_)			31.45 ± 1.78 (a_1_)	
Δ (%)		+29.77			+17.53	
Type A crystallinity (%)						
Before exposure (t = 0 s)		17.06 ± 1.06 (b_2_)			21.94 ± 1.18 (a_2_)	
After exposure (t = 220 s)		21.53 ± 1.05 (b_1_)			25.07 ± 1.64 (a_1_)	
Δ (%)		+26.16			+14.29	
Type V crystallinity (%)						
Before exposure (t = 0 s)		3.42 ± 0.16 (b_2_)			4.83 ± 0.30 (a_2_)	
After exposure (t = 220 s)		5.06 ± 0.28 (b_1_)			6.39 ± 0.36 (a_1_)	
Δ (%)		+47.73			+32.20	

Results are expressed as mean ± standard deviation (*n* = 3); WWF—whole wheat flour; BWF—black wheat flour; Δ (%)—variation relative to the initial time point (t = 0 s). Values followed by different letters (a, b) within the same row indicate statistically significant differences (*p* < 0.05), while values marked with different numbers (1, 2) within the same column denote statistically significant differences (*p* < 0.05).

**Table 3:** Morphology of starch granules isolated from whole and black wheat flour samples, analyzed comparatively before and after MW exposure.

Comparison	SEM	Granule Shape and Size	Surface, Morphology and Texture	Granule Aggregation and Packing	Structural Integrity and Stability	Potential Techno-Functional Implications
Untreated whole wheat	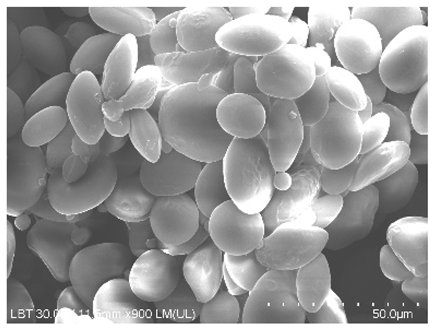 WWF 0 s	Granules exhibit a mostly round to oval shape, typical of native wheat starch. The size distribution is relatively uniform, with minimal deformation.	The surface appears smooth, with well-defined, intact granule boundaries. No visible cracks, pores, or surface roughness are present, indicating high structural integrity.	Granules are loosely packed and distinctly separate from each other. There is minimal clustering, indicating strong individual granule integrity.	Granules are well-preserved with clear, intact boundaries. No visible signs of breakdown, fragmentation, or swelling.	Native wheat is suitable for applications requiring intact starch properties, such as baking, pasta, and thickening agents.
Microwave treated whole wheat for 120 s	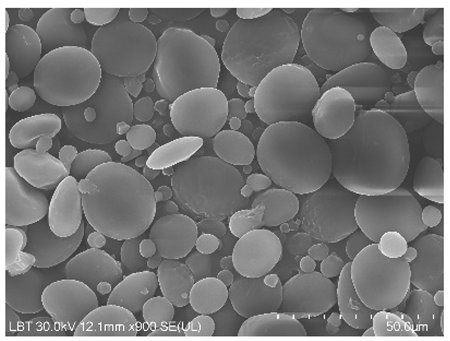 WWF 120 s	The granules appear slightly deformed due to heat exposure. Some granules have become more elongated or irregular in shape. More variation in granule size is observed, with some granules merging or breaking apart.	The surface is roughened with small indentations, fissures, and possible cracks. Some granules exhibit a pitted or wrinkled texture, likely due to moisture loss and expansion-contraction effects during heating.	Granules show increased aggregation, with some forming clusters. Some of them appear to be partially fused or adhered to each other, which suggests partial gelatinization or thermal damage.	Some granules show signs of swelling, deformation, or partial breakdown. A few granules have fragmented, likely due to the rapid heat treatment. The presence of surface cracks and fissures suggests a loss of mechanical strength.	Increased surface roughness and partial granular fissuring provide a larger surface area accessible for hydration and solvent penetration. The compromised granule envelope facilitates faster water uptake during thermal processing, modifying paste viscosity and pasting behavior. Suitable for food matrices requiring rapid cold or hot hydration, such as instant dry mixes, extruded snacks, and composite bakery formulations.
Untreated black wheat	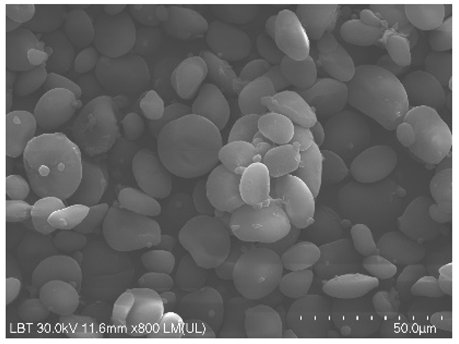 BWF 0 s	Mostly round to oval granules with well-defined edges. Granule size varies within a normal range for starch.	Smooth granule surfaces with minimal visible damage.	Well-separated granules with minimal clustering.	High structural integrity, maintaining the natural starch granule form.	Well-preserved granular integrity characterized by smooth, continuous surfaces free of cracks or thermal cavities. The intact physical barrier limits premature swelling and leaching below gelatinization temperatures. Ideal for conventional breadmaking and pasta processing where standard dough gluten–starch structural integrity and controlled hydration are required.
Microwave treated black wheat for 120 s	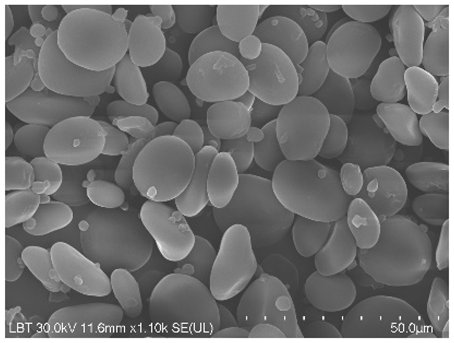 BWF 120 s	Some granules appear deformed or slightly swollen. Increased granule fusion and irregular shapes are observed.	Roughened surfaces with cracks and small indentations due to heat treatment.	Increased granule aggregation, with some fused or partially broken granules.	Partial breakdown of structural integrity, indicating starch gelatinization or early degradation.	Partial granular indentation and surface pore expansion facilitate intra-granular water diffusion, promoting accelerated swelling and lower pasting temperatures. These microstructural modifications favor enhanced cold-water swelling capacity and matrix-binding efficiency, making it suitable for functional thickeners, formulated sauce stabilizers, and specialty baked goods.

**Table 4:** Kinetic parameters of starch hydrolysis, nutritional fractions, and in vitro digestibility indices for whole wheat flour (WWF) and black wheat flour (BWF) subjected to microwave thermal treatment.

Sample	C_∞_ (%)	k (min^−1^)	R^2^	AUC (% · min)	eGI (%)	HI (%)	ΔHI (%)	RDS (20 min) (%)	SDS (20–120 min) (%)	RS (>180 min) (%)
WWF 0	70.25 ± 3.15 (c2)	0.0061 ± 0.0018 (c1)	0.99	4941.01 ± 140.32 (e1)	94.61 ± 1.56 (c1)	100.00 ± 2.84 (e1)	-	8.09 ± 0.28 (d2)	29.24 ± 0.85 (d1)	62.67 ± 1.45 (ab2)
WWF 40	87.84 ± 3.28 (ab1)	0.0048 ± 0.0019 (c1)	0.99	5232.12 ± 167.95 (de2)	97.84 ± 1.87 (bc2)	105.89 ± 3.40 (de2)	5.89 ± 0.34 (d2)	11.35 ± 0.36 (c2)	23.99 ± 0.74 (e2)	64.66 ± 1.55 (a1)
WWF 80	90.50 ± 4.21 (a1)	0.0052 ± 0.0011 (c2)	0.99	5711.83 ± 122.80 (cd2)	103.17 ± 1.45 (b2)	115.60 ± 2.65 (cd2)	15.60 ± 0.26 (c2)	8.81 ± 0.25 (d2)	30.14 ± 0.78 (cd2)	61.06 ± 1.35 (b1)
WWF 120	93.53 ± 3.17 (a1)	0.0065 ± 0.0012 (c2)	0.98	5834.08 ± 201.28 (c2)	104.53 ± 2.23 (b2)	118.07 ± 4.07 (c2)	18.07 ± 0.47 (c2)	7.96 ± 0.26 (d2)	32.28 ± 0.95 (bc2)	59.76 ± 1.62 (b1)
WWF 160	80.76 ± 3.73 (b2)	0.0242 ± 0.0032 (b2)	0.98	11,246.62 ± 294.66 (a1)	164.67 ± 3.36 (a1)	227.62 ± 6.12 (a1)	127.62 ± 5.12 (a1)	34.57 ± 0.98 (b1)	40.03 ± 1.15 (a1)	25.39 ± 0.72 (c2)
WWF 200	67.03 ± 2.12 (c2)	0.0441 ± 0.0105 (a1)	0.95	10,545.83 ± 329.03 (b2)	156.89 ± 3.65 (a1)	213.43 ± 6.65 (b2)	113.43 ± 4.65 (b2)	37.08 ± 1.12 (a1)	34.45 ± 0.92 (b2)	28.56 ± 0.81 (c1)
BWF 0	74.50 ± 3.20 (d1)	0.0055 ± 0.0008 (d2)	0.95	4897.72 ± 119.99 (f2)	94.61 ± 1.35 (f1)	100.00 ± 2.45 (f1)	-	10.73 ± 0.31 (f1)	24.04 ± 0.68 (d2)	65.23 ± 1.58 (a1)
BWF 40	82.40 ± 3.15 (cd2)	0.0068 ± 0.0011 (cd1)	0.98	6277.57 ± 194.60 (e1)	110.08 ± 2.18 (e1)	128.17 ± 3.97 (e1)	28.17 ± 0.39 (e1)	13.58 ± 0.42 (e1)	40.22 ± 1.18 (b1)	46.20± 1.25 (b2)
BWF 80	85.80 ± 4.02 (bc2)	0.0075 ± 0.0012 (cd1)	0.96	6969.71 ± 158.91 (d1)	117.84 ± 1.78 (d1)	142.31 ± 3.24 (d1)	42.31 ± 0.32 (d1)	15.36 ± 0.45 (d1)	52.22 ± 1.42 (a1)	32.42 ± 0.85 (e2)
BWF 120	88.50 ± 3.50 (abc2)	0.0113 ± 0.0018 (bc1)	0.96	9122.64 ± 250.87 (c1)	141.97 ± 2.81 (c1)	186.26 ± 5.12 (c1)	86.26 ± 0.51 (c1)	19.41 ± 0.58 (c1)	34.68 ± 0.95 (c1)	45.91 ± 1.28 (b2)
BWF 160	92.10 ± 3.71 (ab1)	0.0142 ± 0.0022 (ab1)	0.96	10,595.49 ± 353.89 (b2)	158.48 ± 3.96 (b2)	216.34 ± 7.22 (b2)	116.34 ± 4.22 (b2)	22.30 ± 0.68 (b2)	36.25 ± 1.05 (c2)	41.44 ± 1.15 (c1)
BWF 200	95.30 ± 3.07 (a1)	0.0171 ± 0.0025 (a2)	0.98	11,837.55 ± 258.06 (a1)	172.40 ± 2.89 (a1)	241.70 ± 5.27 (a1)	141.70 ± 4.27 (a1)	25.08 ± 0.72 (a2)	36.71 ± 0.98 (c1)	38.21 ± 0.95 (d1)

Values represent the mean ± standard deviation of triplicate determinations (*n* = 3). Different lowercase letters (a–f) within the same column for the same flour across different microwave exposure times indicate statistically significant differences (*p* < 0.05) according to Tukey’s HSD test. Different numbers (1, 2) within the same column comparing different flours at the same exposure time indicate statistically significant differences (*p* < 0.05) (Tukey’s test). WWF—whole wheat flour; BWF—black wheat flour; 0–200 indicates the duration of microwave thermal exposure (seconds). Kinetic parameters (*C*_∞_−estimated maximum hydrolyzable starch percentage at equilibrium, %; k−hydrolysis rate constant, min^−1^; and *R*^2^−coefficient of determination) were obtained by non-linear first-order fitting according to the equation: *y* = *C*_∞_ ⋅ (1 − *e*^−*k*⋅*t*^). Starch nutritional fractions were classified as: RDS (%)—rapidly digestible starch (hydrolyzed within the first 20 min); SDS (%)—slowly digestible starch (hydrolyzed between 20 and 120 min); RS (%)—resistant starch (unhydrolyzed at 120 min); AUC—area under the hydrolysis curve integrated over the 0–180 min (% · min); HI (%)—hydrolysis index relative to the corresponding native control flour of each series (0 s = 100%); ΔHI−change in the hydrolysis index relative to the reference at t = 0 s (%); eGI—estimated glycemic index.

**Table 5:** FTIR protein secondary structure, evolution of free –SH, total –SH, and –SS groups in wheat protein isolates from wheat samples subjected to microwave treatment.

Protein Secondary Structure Obtained by FTIR Data Processing							
**Exposure Time (s)**	Parameter			WWF		BWF	
Before MW exposure (t = 0 s)	α-helix (%)			24.82 ± 0.32 (a_2_)		22.17 ± 0.15 (b_2_)	
	β-sheet (%)			37.04 ± 0.44 (a_1_)		37.97 ± 0.44 (a_1_)	
	β-turn (%)			22.32 ± 0.18 (a_2_)		21.24 ± 0.28 (b_2_)	
	random coil (%)			15.83 ± 0.14 (b_1_)		18.61 ± 0.18 (a_1_)	
	Sum (α-helix + β-sheet + β-turn)			84.18 ± 1.08 (a_1_)		81.38 ± 0.87 (b_1_)	
After MW exposure (t = 220 s)	α-helix (%)			25.55 ± 0.23 (a_1_)		23.87 ± 0.42 (b_1_)	
	β-sheet (%)			32.68 ± 0.17 (a_2_)		31.49 ± 0.37 (b_2_)	
	β-turn (%)			30.14 ± 0.52 (a_1_)		26.38 ± 0.11 (b_1_)	
	random coil (%)			11.64 ± 0.28 (b_2_)		18.25 ± 0.23 (a_1_)	
	Sum (α-helix + β-sheet + β-turn)			88.37 ± 0.92 (a_2_)		81.74 ± 0.90 (b_1_)	
Variation after exposure Δ (%)	α-helix (%)			2.96		7.67	
	β-sheet (%)			−11.77		−17.06	
	β-turn (%)			35.05		24.15	
	Random coil (%)			−26.50		−1.91	
Parameter	WWF protein isolate			BWF protein isolate			
	Before MW exposure (t = 0 s)	After 120 s of MW exposure	After 220 s of MW exposure	Before MW exposure (t = 0 s)	After 120 s of MW exposure		After 220 s of MW exposure
Free—SH (μmol/g)	2.44 ± 0.01 (a_1_)	0.86 ± 0.03 (c_2_)	1.00 ± 0.02 (b_1_)	4.78 ± 0.06 (b_1_)	2.06 ± 0.07 (c_1_)		8.39 ± 0.05 (a_1_)
Δ (%)	-	−64.75	−59.01	-	−56.90		+75.52
Total—SH (μmol/g)	4.48 ± 0.03 (c_2_)	5.34 ± 0.04 (b_2_)	11.61 ± 0.08 (a_2_)	14.39 ± 0.08 (b_1_)	10.48 ± 0.24 (c_1_)		19.30 ± 0.01 (a_1_)
Δ (%)	-	+19.19	+159.15	-	−27.17		+34.12
-SS bonds (μmol/g)	1.02 ± 0.02 (c_2_)	2.24 ± 0.03 (b_2_)	5.30 ± 0.04 (a_2_)	4.80 ± 0.05 (b_1_)	4.21 ± 0.15 (c_1_)		5.46 ± 0.10 (a_1_)
Δ (%)	-	+54.46	+419.60	-	−12.29		+13.75

Results are expressed as mean ± standard deviation (n = 3). Values followed by different letters (a, b, c) within the same row indicate statistically significant differences (*p* < 0.05) for the same sample at different exposure times, while values marked with different numbers (1, 2) within the same row denote statistically significant differences (*p* < 0.05) between different samples at the same exposure time. WWF—whole wheat flour; BWF—black wheat flour; Δ (%)—variation relative to the initial time point (t = 0 s).

## Data Availability

The original contributions presented in the study are included in the article; further inquiries can be directed to the corresponding author.

## Supplementary Materials

The following supporting information can be downloaded at: https://www.mdpi.com/article/10.3390/foods15193537/s1, Figure S1: FTIR starch and protein deonvolution; Figure S2: XRD deconvolution process; Figure S3: Mixolab rheological parameters; Figure S4: Pearson Correlations; Figure S5: First-order non-linear fitted curves of in-vitro starch hydrolysis kinetics for whole wheat flour (WWF) and black wheat flour (BWF) subjected to different microwave exposure durations (0-200 s); Table S1: Baseline proximate composition and phytochemical profile of native whole wheat flour (WWF) and black wheat flour (BWF); Table S2: Thermal evolution and moisture loss kinetics of whole wheat flour (WWF) and black wheat flour (BWF) during microwave treatment.
