# Supplementary material for: Impact of Microwave Treatment on Protein and Starch Characteristics of Black Wheat and Whole Wheat (T. aestivum L.) Flour
Source: Foods. 2026 Oct 3;15(19):3537. doi: 10.3390/foods15193537 (PMC13665050; doi:10.3390/foods15193537)
Supplement: Supplementary file 1 [file foods-15-03537-s001.zip › foods-4576744-supplementary.pdf]

Supplementary Material

Impact of Microwave Treatment on Protein and Starch Characteristics of Black Wheat and Whole Wheat *(T. aestivum L.)* Flour

A. M. Uivarasan[1*], L. Mihaly Cozmuta[1], A. Peter[1], C. Nicula[1], Z. Szakács[1], E. Alexa[2], S. Dossa[2], M. Muresan-Pop[3,4], K. Magyari[3,4], M. Baia[3,5], I. Botiz[3,5], L. Barbu-Tudoran[6,7], Z. Vosgan[1], B. Mihalescu[1], C. Pintilii[1], L. Pop[1], A. Babiciu[1], M. Tarau[1], A. Cosma[1], M. Marcus[1], A. Mihaly Cozmuta[1]

[1]Technical University of Cluj Napoca, North University Center of Baia Mare, Victoriei Str. 76, Baia Mare, Romania;

[2] Faculty of Food Engineering, University of Life Sciences “King Michael I” from Timisoara, Romania;

[3]Nanostructured Materials and Bio-Nano-Interfaces Center, Interdisciplinary Research Institute on Bio-Nano-Sciences, Babes-Bolyai University, T. Laurian 42, 400271, Cluj-Napoca, Romania

[4]INSPIRE Research Platform InfoBioNano4Health & Biomedical Imaging, Babeș Bolyai University, Arany Janos 11, 400084 Cluj-Napoca, Romania

[5]Faculty of Physics, Babes-Bolyai University, M. Kogălniceanu 1, 400347, Cluj-Napoca, Romania

[6]Faculty of Biology and Geology, Babes-Bolyai University, Clinicilor Str. 5-7, 400006, Cluj-Napoca, Romania

[7]National Institute for Research and Development of Isotopic and Molecular Technologies, Donath Str. 67-103, 400293 Cluj-Napoca, Romania

*uivarasanalexandra@yahoo.com

S2. Materials and Methods

S2.1. Deconvolution of FTIR spectra and XRD diffractograms

A) Changes in the ordered structure of starch were investigated in the spectral ranges of 900–1200 cm^{-1} and 1550–1700 cm^{-1} [1]. To enable accurate quantification, the FTIR spectra were first normalized, bringing all absorbance values into the 0-1 range to eliminate differences caused by sample thickness or concentration. The normalized spectra were subsequently smoothed using the Savitzky-Golay algorithm (third-order polynomial, 25-point window), which reduces random noise while it also preserves peak shape and intensity. After smoothing, a baseline correction was applied, limited to the starch-specific region, to further isolate the structural features of interest.

The next step consisted in identifying characteristic wavenumbers associated with crystalline and amorphous starch domains. These positions were determined from the minima of the second-order derivative of the spectra, calculated using the same Savitzky-Golay parameters (third-order polynomial, 25-point window). The derivative approach allowed accurate localization of overlapping absorption bands by highlighting inflection points corresponding to hidden or weak spectral features.

Each of the ten minima identified were assigned as the mean of ten Gaussian distributions, initialized with a multiplication factor of 2 and a standard deviation of 5. Gaussian deconvolution was then performed on the starch-specific region, enabling the separation of individual overlapping bands and quantification of their relative contributions. This approach provided the basis for estimating the short-range molecular order index, by comparing the relative areas of crystalline-associated versus amorphous-associated bands.

Figure S1. illustrates the sequential steps of the deconvolution process for starch-specific region: **(a)** raw FTIR spectrum prior to normalization; **(b)** spectrum after normalization; **(c)** normalized spectrum before smoothing; **(d)** normalized spectrum after Savitzky-Golay filtering; **(e)** smoothed spectrum prior to baseline correction; **(f)** smoothed spectrum after baseline correction; **(g)** second-order derivative profile obtained in the starch-specific region; and **(h)** Gaussian peak initialization at derivative minima used for band fitting.

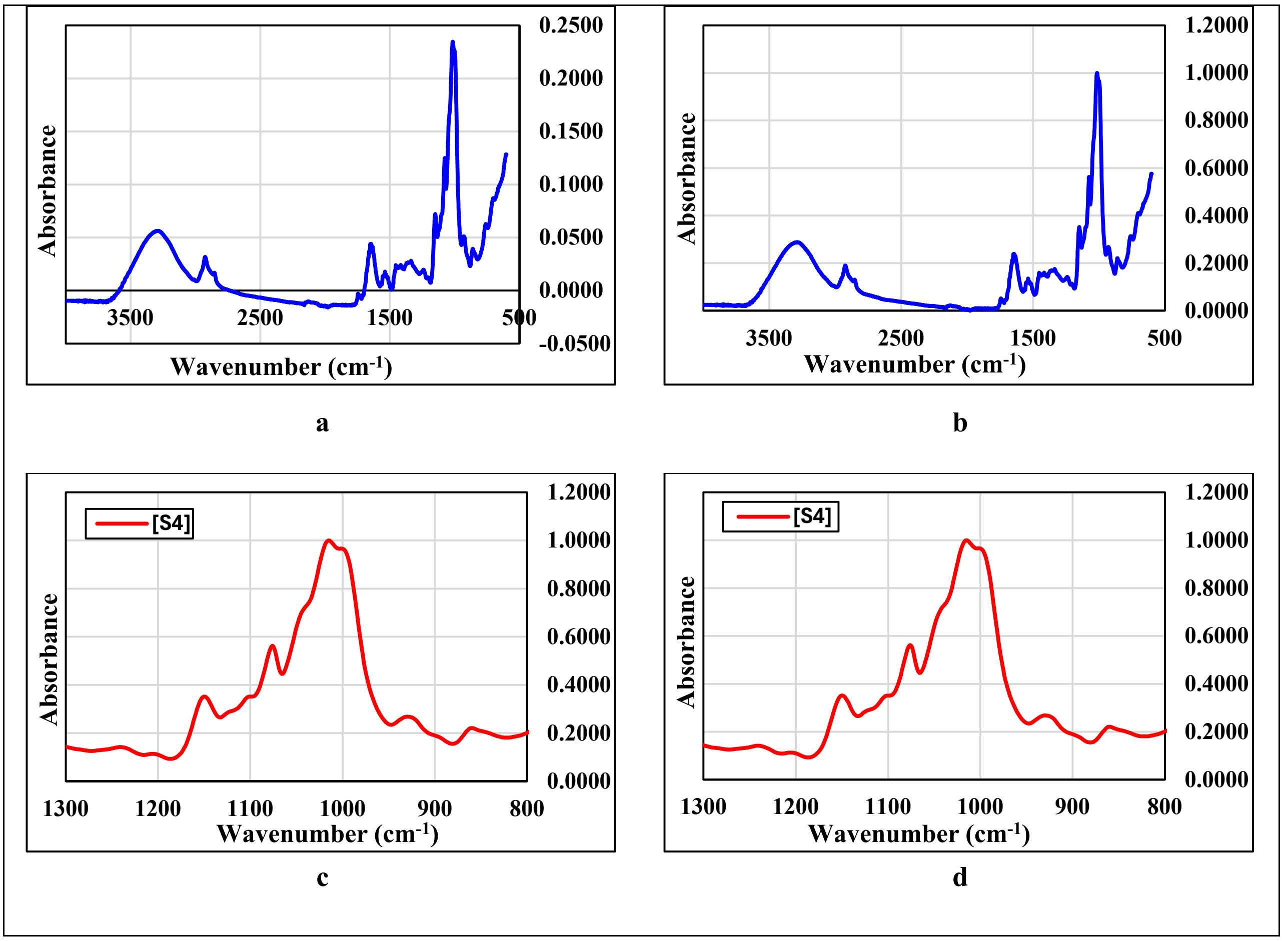

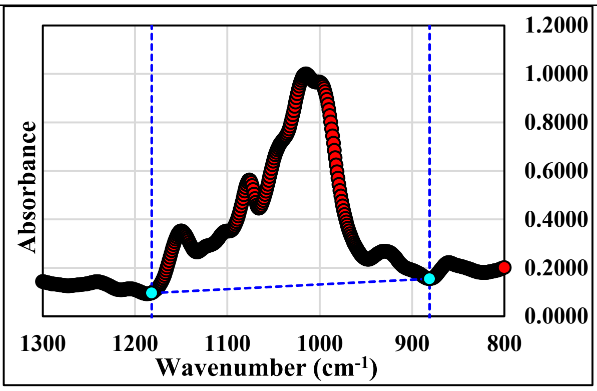
Absorbance
1.2000
1.0000
0.8000
0.6000
0.4000
0.2000
0.0000
1300
1200
1100
1000
900
800
Wavenumber (cm-1)

e

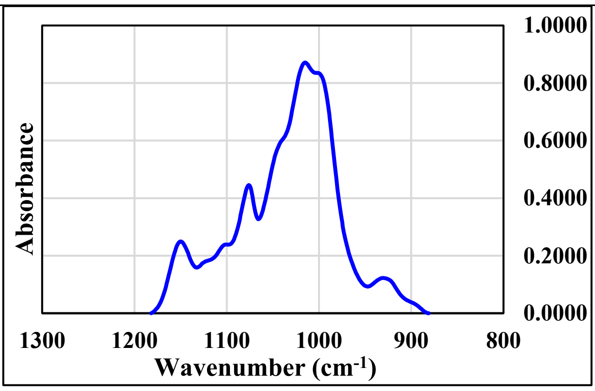
Absorbance
1.0000
0.8000
0.6000
0.4000
0.2000
0.0000
1300
1200
1100
1000
900
800
Wavenumber (cm-1)

f

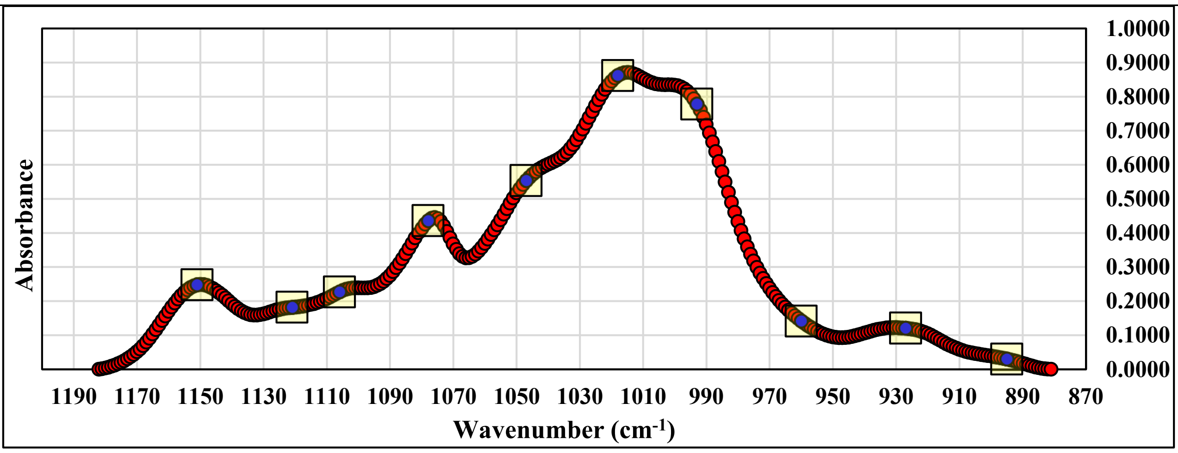
Absorbance
1.0000
0.9000
0.8000
0.7000
0.6000
0.5000
0.4000
0.3000
0.2000
0.1000
0.0000
1190 1170 1150 1130 1110 1090 1070 1050 1030 1010 990 970 950 930 910 890 870
Wavenumber (cm-1)

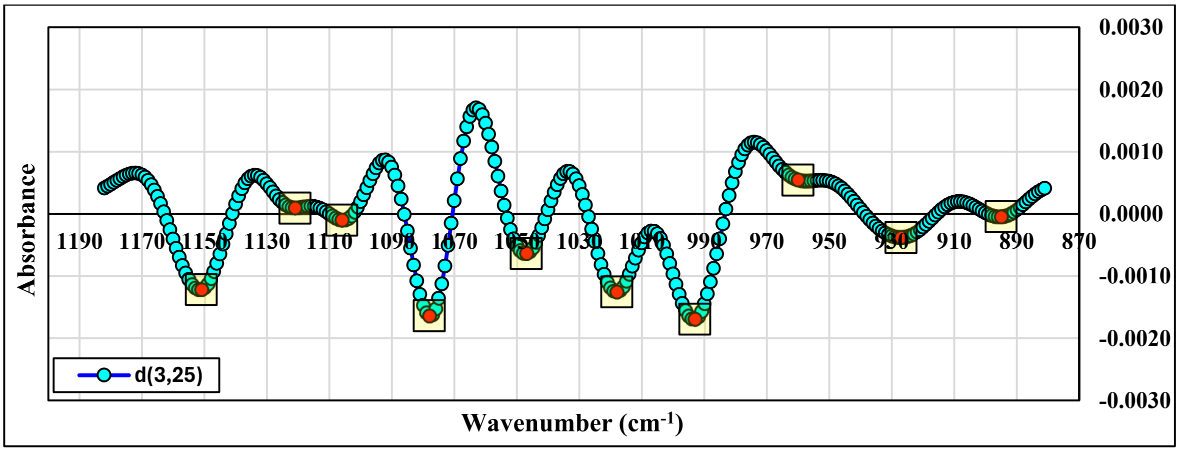
Absorbance
0.0030
0.0020
0.0010
0.0000
-0.0010
-0.0020
-0.0030
1190 1170 1150 1130 1110 1090 1070 1050 1030 1010 990 970 950 930 910 890 870
d(3,25)
Wavenumber (cm-1)

g

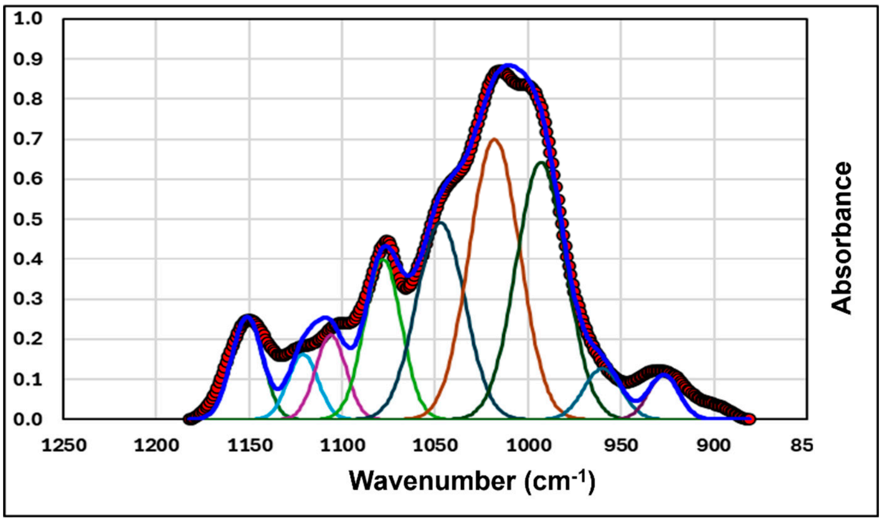

h

Figure S1. FTIR Starch and protein deconvolution

For each wavenumber, the sum of the ten Gaussian distributions considered was calculated, and the squared differences between this sum and the experimental starch spectrum were determined. The sum of squared differences was used as the objective function for spectral deconvolution, performed with the Solver tool (Microsoft Office Excel). Optimization was carried out using the nonlinear GRG (Generalized Reduced Gradient) model, with the convergence criterion set to 0.001, and the process was iterated until error minimization was achieved.

B) The secondary structure of proteins was determined by deconvolution of FTIR spectra in the amide I region (1700–1550 cm^{-1}), which is characteristic of protein vibrations. The individual components of the deconvoluted curves were quantified by assigning spectral contributions to specific secondary structures: *β-sheet* (ordered structures, 1636–1615 cm^{-1} for the low-frequency region and 1700–1682 cm^{-1} for the high-frequency region), *random coil* (disordered structures, 1645–1637 cm^{-1}), *α-helix* (densely ordered structures, 1663–1646 cm^{-1}), and *β-turn* (extended ordered structures, 1681–1664 cm^{-1}) [2].

The algorithm applied was similar to that used for starch crystallinity determination, involving Gaussian deconvolution of the specific spectral domain and calculation of the respective peak areas (Figure S1 i-l). This approach enabled the quantification of the relative proportions of the main protein conformations: α-helix, β-sheet, β-turn, and random coil.

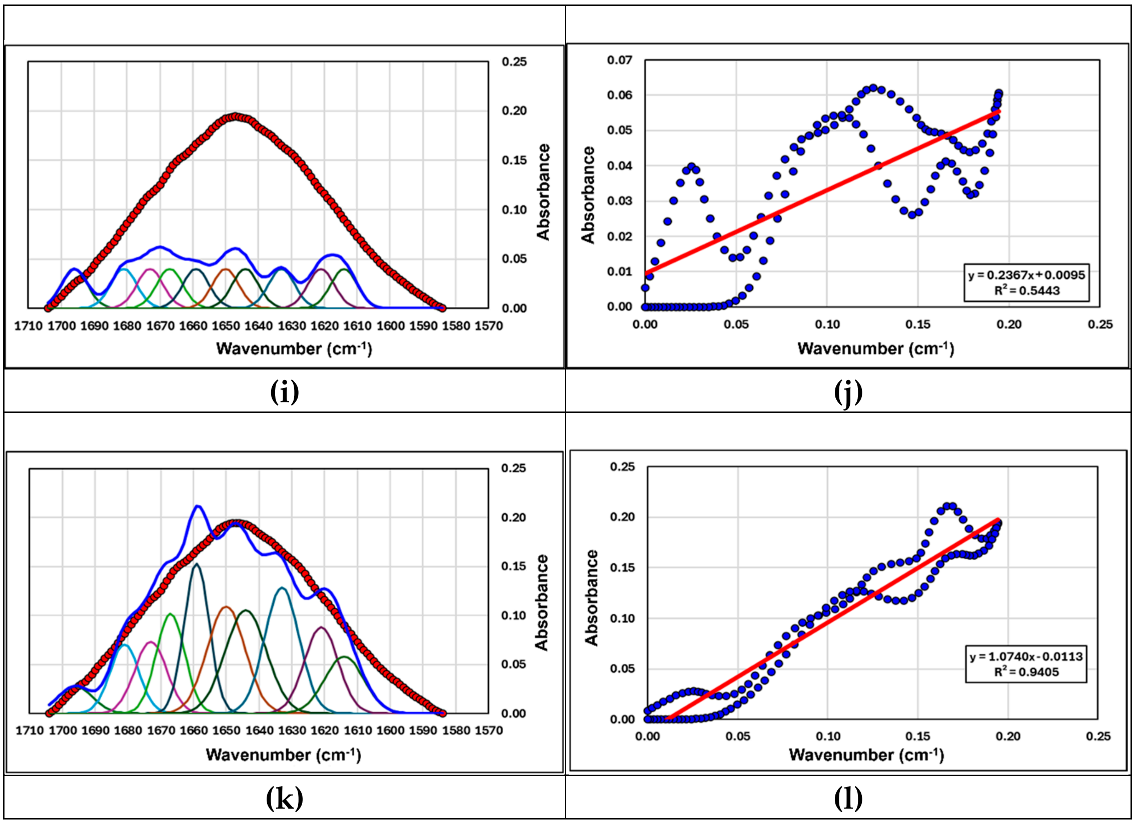

Figure S1. FTIR Starch and protein deconvolution

The evaluation of protein secondary structures was carried out following the methodology of Zhao et al. [2], using the reference spectral ranges of the amide I band as follows:

(i) 1636–1615 cm^{-1} and 1700–1682 cm^{-1} corresponding to β-sheet structures;

(ii) 1645–1637 cm^{-1} corresponding to random coil structures;

(iii) 1663–1646 cm^{-1} attributed to α-helix conformations;

(iv) 1681–1664 cm^{-1} corresponding to β-turn structures.

The Gaussian peak areas, identified based on the minima of the second-order derivative within these spectral domains, were used to calculate the relative proportions of the different protein secondary structure conformations.

S2.2. XRD diffractogram deconvolution and crystallinity assessment

The deconvolution process applied to the XRD diffractograms is illustrated in Figure S2, following a computational workflow similar to that employed for FTIR spectral deconvolution.

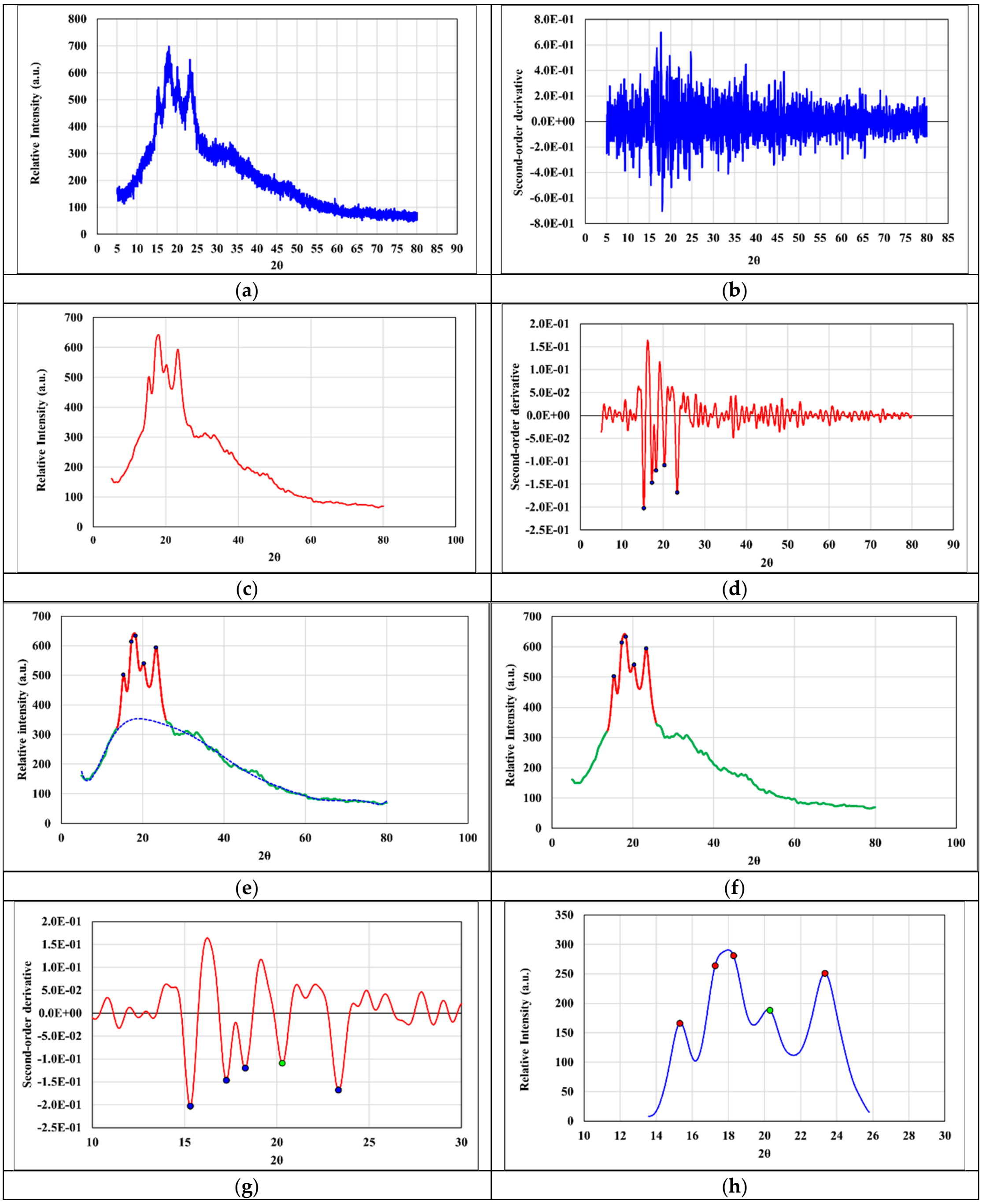

Figure S2. XRD deconvolution process: **(a)** - Original X-ray diffraction pattern of black wheat starch; **(b)** - Second-order derivative of the original X-ray diffraction pattern; **(c)** - X-ray diffraction pattern after application of the Savitzky–Golay filter; **(d)** - Second-order derivative of the Savitzky–Golay filtered diffraction pattern; **(e)** - Delimitation of crystalline and amorphous domains in the diffraction pattern: crystalline peaks (red) and baseline determined from amorphous regions (green); **(f)** - Separation of amorphous (below baseline, blue) and crystalline

(above baseline) areas in the XRD profile; **(g)** - Baseline fitting and **(h)** - identification of diffraction maxima characteristic of A- and Vh-type crystallization forms;

To resolve overlapping crystalline reflections and decouple the amorphous background, diffraction patterns were processed in OriginPro software (OriginLab Corp., Northampton, MA, USA). High-frequency instrumental noise was reduced using a Savitzky-Golay smoothing filter (polynomial order 3, 25-point window) without distorting peak geometry or altering diffraction angular positions. Peak center coordinates were pinpointed via second-order derivative analysis ($d^2 I/d\theta^2$), resolving closely spaced reflection maxima characteristic of A-type starch polymorphs ($2\theta \approx 15^{o}, 17.1^{o}, 17.9^{o}, 23.1^{o}$) and Vh-type amylose-lipid complexes ($2\theta \approx 13.2^{o}, 19.8^{o}$).

A non-linear continuous baseline connecting the lowest intensity points was fitted to separate the diffuse amorphous halo from the overlying crystalline diffraction domain. Non-linear curve fitting of the crystalline peaks above the baseline was performed using Gaussian/pseudo-Voigt functions until mathematical convergence ($R^2 >$ 0.99). Relative crystallinity (RC, %) was quantified as the ratio of the total integrated crystalline peak area (A_c) to the total area of the diffractogram ($A_c + A_a$):

$$RC\ (\%) = \frac{A_c}{A_c + A_a} \times 100 \qquad \textbf{(S1)}$$

where: A_c is the integrated area of all resolved crystalline peaks and A_a represents the integrated area of the underlying amorphous halo.

S2.3. Determination of total anthocyanins

Total anthocyanin content was determined using an adapted spectrophotometric method according to Sytar et al. [3]. Briefly, an accurately weighed flour sample was extracted with 3.0 mL of acidified methanol (1% HCl, v/v) for 24 h in the dark at 4 °C, with periodic agitation. Subsequently, 2.0 mL of distilled water and 4.8 mL of chloroform were added to the extract, followed by thorough mixing. After centrifugation of the biphasic mixture at 5,000 × g for 15 min, the upper aqueous-methanolic phase was collected, and its absorbance was measured at 530 nm and 657 nm. Total anthocyanin concentration (expressed as mg/100 g dry weight) was calculated using the following equation:

$$TAC = (OD_{530} - 0.25 \times OD_{657}) \times \frac{TV}{DW} \times 100 \qquad \textbf{(S2)}$$

where OD_{530} and OD_{657} represent the optical density at the respective wavelengths, TV is the total volume of the extract (mL), and DW is the dry weight of the sample (g).

S2.4. Determination of individual phenolic compounds

Chromatographic quantification of individual phenolic compounds was performed on a Shimadzu HPLC platform (Kyoto, Japan) equipped with an SPD-10A UV detector and an LC-MS 2010 mass spectrometer, adapting the chromatographic conditions described by Plustea et al. [4] and Dragomir et al. [5] as part of our baseline flour characterization [**15**, in the main text]. Compound separation was carried out on a reversed-phase NUCLEODUR C18 Gravity SB column (150 × 2 mm, 5 μm particle size; Macherey-Nagel) maintained at a constant temperature of 35 °C.

The elution system utilized a binary mobile phase consisting of acidified water adjusted to pH 3.0 with formic acid (solvent A) and acetonitrile acidified to pH 3.0 with formic acid (solvent B), delivered at a flow rate of 0.2 mL/min. The multi-stage gradient profile was programmed as follows: isocratic 5% B (0-20 min); linear gradient from 5% to 40% B (20.01-50 min); rapid ramp from 40% to 95% B (50.01-55 min); and an isocratic plateau at 95% B (55.01-60 min) for column washing.

Spectral data were acquired at 280 nm and 320 nm, with analyte quantification conducted at 280 nm. Targeted polyphenols were identified and calculated against five-point linear calibration curves of authentic reference standards across a range of 20-50 μg/mL. Concentrations were expressed as mg/kg dry weight, and all chromatographic determinations were performed in triplicate [6].

S3. Results and discussion

S3.1. Additional rheological (Mixolab) parameters

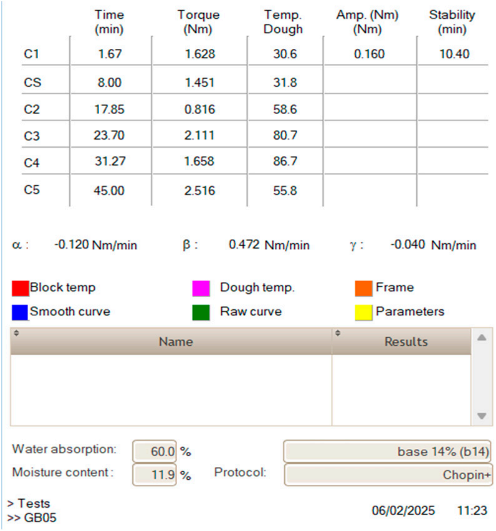

	Time (min)	Torque (Nm)	Temp. Dough	Amp. (Nm) (Nm)	Stability (min)
C1	1.67	1.628	30.6	0.160	10.40
CS	8.00	1.451	31.8		
C2	17.85	0.816	58.6		
C3	23.70	2.111	80.7		
C4	31.27	1.658	86.7		
C5	45.00	2.516	55.8		

α : -0.120 Nm/min β : 0.472 Nm/min γ : -0.040 Nm/min

Block temp Dough temp. Frame
Smooth curve Raw curve Parameters

Name	Results

Water absorption: 60.0 % base 14% (b14)
Moisture content : 11.9 % Protocol: Chopin+
> Tests
>> GB05
06/02/2025 11:23

WWF 0s 0%

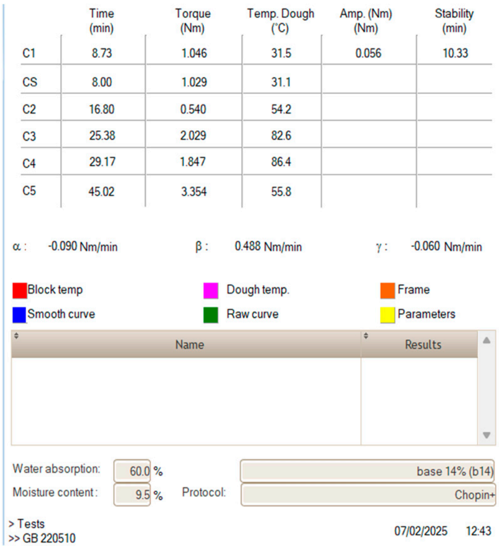

	Time (min)	Torque (Nm)	Temp. Dough (°C)	Amp. (Nm) (Nm)	Stability (min)
C1	8.73	1.046	31.5	0.056	10.33
CS	8.00	1.029	31.1		
C2	16.80	0.540	54.2		
C3	25.38	2.029	82.6		
C4	29.17	1.847	86.4		
C5	45.02	3.354	55.8		

α : -0.090 Nm/min β : 0.488 Nm/min γ : -0.060 Nm/min

Block temp Dough temp. Frame
Smooth curve Raw curve Parameters

Name	Results

Water absorption: 60.0 % base 14% (b14)
Moisture content : 9.5 % Protocol: Chopin+
> Tests
>> GB 220510
07/02/2025 12:43

BWF 220s 10%

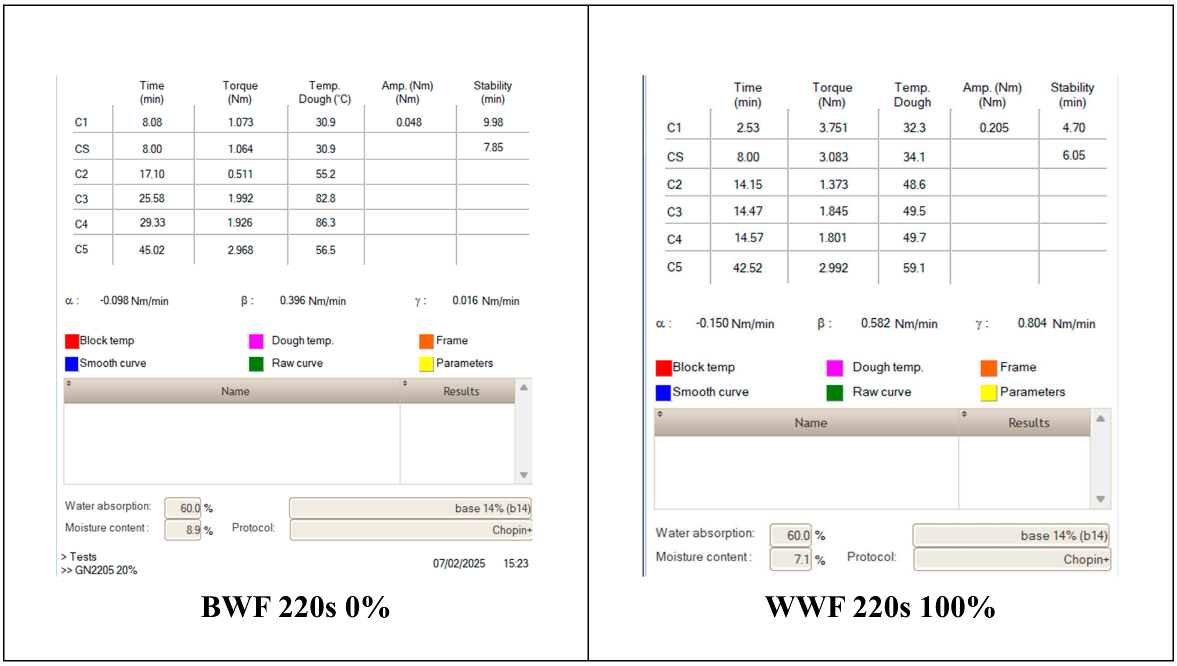

Figure S3. Mixolab rheological parameters

S3.2 Pearson correlations between key parameters

Parameter	XRD total starch	XRD type A starch	XRD type Vh starch	FTIR RCD (%)	Mixolab C1	Mixolab C2	Mixolab C3	Mixolab C4	Mixolab C5
XRD total starch	1	0.98	0.71	0.74	-0.43	-0.43	0.86	0.8	0.77
XRD type A starch	0.98	1	0.54	0.58	-0.23	-0.23	0.73	0.66	0.61
XRD type Vh starch	0.71	0.54	1	1	-0.94	-0.94	0.97	0.98	0.99
FTIR RCD (%)	0.74	0.58	1	1	-0.92	-0.92	0.98	0.99	1
Mixolab C1	-0.43	-0.23	-0.94	-0.92	1	1	-0.83	-0.88	-0.91
Mixolab C2	-0.43	-0.23	-0.94	-0.92	1	1	-0.83	-0.88	-0.91
Mixolab C3	0.86	0.73	0.97	0.98	-0.83	-0.83	1	0.99	0.99
Mixolab C4	0.8	0.66	0.98	0.99	-0.88	-0.88	0.99	1	0.99
Mixolab C5	0.77	0.61	0.99	1	-0.91	-0.91	0.99	0.99	1

-1   0   1

Figure S4. Pearson Correlations

S3.3. Baseline physicochemical and phenolic profile of raw materials

Table S1. summarizes the foundational proximate composition and preliminary phytochemical properties of the unheated native whole wheat flour (WWF) and black wheat flour (BWF) used in this work. Complete experimental details and extended datasets regarding individual mineral distribution, HPLC polyphenolic profile, and thermal properties are reported in our companion baseline study ([15] in main text).

Table S1. Baseline proximate composition and phytochemical profile of native whole wheat flour (WWF) and black wheat flour (BWF)

Parameter	WWF (Control)	BWF (Black wheat flour)
Moisture (%)	8.40 ± 0.07^{b}	11.36 ± 0.13^{a}
Crude Protein (%)	11.99 ± 0.23^{b}	13.55 ± 0.44^{a}
Total Lipids (%)	1.53 ± 0.09^{b}	2.46 ± 0.20^{a}
Ash (%)	1.59 ± 0.04^{b}	1.74 ± 0.03^{a}
Crude fiber (g/100g)	3.35 ± 0.15^{a}	2.16 ± 0.16^{b}
Available Carbohydrates (g/100g)	73.14 ± 3.15^{a}	68.73 ± 2.01^{b}
Energy (kcal/100g)	360.99 ± 8.17^{a}	355.58 ± 9.20^{a}
**Phytochemical and antioxidant profile**		
Total Phenolics (mg GAE/100g)	110.95 ± 1.20^{b}	259.30 ± 1.63^{a}
Total Flavonoids (mg QE/100g)	150.51 ± 0.83^{a}	88.49 ± 0.62^{b}
Total Anthocyanins (mg/100g)	25.00 ± 0.92^{b}	93.00 ± 1.97^{a}
DPPH Scavenging Activity (%)	9.58 ± 0.34^{b}	26.56 ± 0.26^{a}
Epicatechin (mg/kg)	184.67 ± 2.31^{b}	201.30 ± 4.17^{a}
Resveratrol (mg/kg)	420.26 ± 5.01^{b}	663.29 ± 14.39^{a}

Values are expressed as mean ± standard deviation ($n \geq 3$). Different superscript letters in the same row indicate statistically significant differences between samples ($p < 0.05$) Tukey's HSD test). GAE - gallic acid equivalents; QE - quercetin equivalents. Baseline data adapted from companion study [15] in the main text, supplemented with baseline determination of total anthocyanins.

S3.4. Thermal history and moisture dynamics during microwave exposure

Table S2. Thermal evolution and moisture loss kinetics of whole wheat flour (WWF) and black wheat flour (BWF) during microwave treatment.

MW exposure time (s)	WWF temperature (°C)	WWF moisture (%)	BWF temperature (°C)	BWF moisture (%)
0	21.00 ± 0.50	8.40 ± 0.07^{a}	21.00 ± 0.50	11.36 ± 0.13^{a}
40	51.90 ± 1.20	6.82 ± 0.12^{b}	50.50 ± 1.40	6.85 ± 0.15^{b}
80	80.80 ± 1.70	2.17 ± 0.08^{c}	78.10 ± 1.90	2.68 ± 0.09^{c}
120	109.30 ± 2.40	0.53 ± 0.05^{d}	105.30 ± 2.60	1.57 ± 0.06^{d}
160	130.90 ± 3.10	< 0.10	126.80 ± 2.80	< 0.10
200	152.10 ± 3.50	< 0.10	150.60 ± 3.20	< 0.10

Values represent the mean ± standard deviation (n = 3). Moisture determinations were performed immediately post-treatment. Surface temperatures were monitored using an infrared thermometer immediately upon treatment cessation. Values < 0.10% represent residual moisture contents below the analytical quantification threshold. Different superscript

letters within each column indicate statistically significant differences across treatment intervals ($p < 0.05$, Tukey's HSD test).

S3.5. In-vitro starch digestion kinetics and nutritional fractions

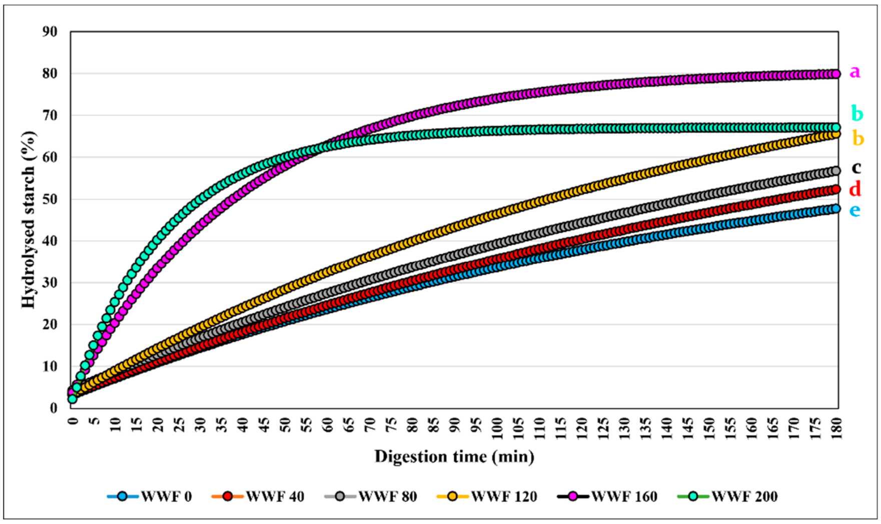

Figure S5. a. First-order non-linear fitted curves of *in vitro* starch hydrolysis kinetics for whole wheat flour (WWF) subjected to different microwave exposure durations (0-200 s). Curves were generated by non-linear regression using the Goñi et al. [7] model ($C_t = C_\infty \cdot [1 - e^{-k \cdot t}]$). Different lowercase letters (a-e) at 180 min indicate statistically significant differences among treatment times ($p < 0.05$, one-way ANOVA, Tukey's HSD post-hoc test).

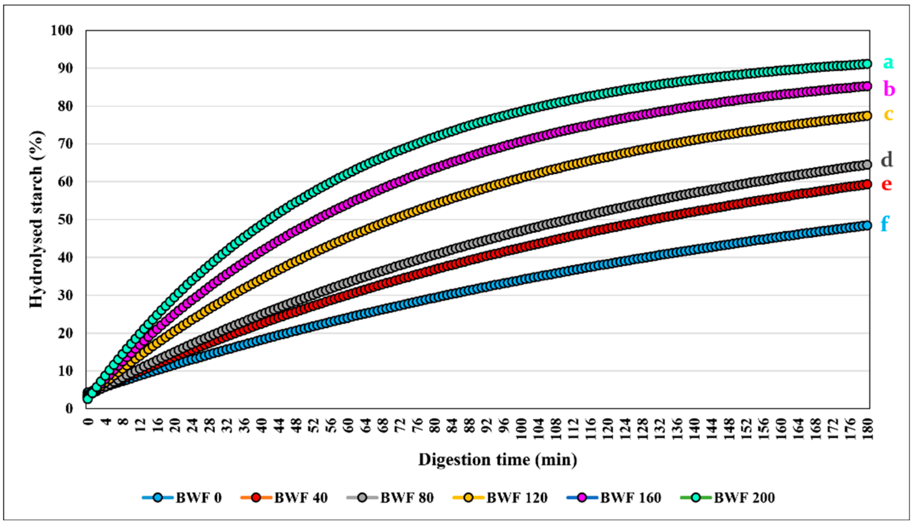

Figure S5.b. First-order non-linear fitted curves of *in-vitro* starch hydrolysis kinetics for black wheat flour (BWF) subjected to different microwave exposure durations (0-200 s). Curves were generated by non-linear regression using the Goñi et al. [7] model ($C_t = C_\infty \cdot [1 - e^{-k \cdot t}]$). Different lowercase letters (a-f) at 180 min

indicate statistically significant differences among treatment times ($p < 0.05$, one-way ANOVA, Tukey's HSD post-hoc test).

References

S1. Wang, Y.; Liu, C.; Lang, H.; Hu, Z.; Wang, X.; Yang, Z.; Wang, Z.; Guo, Z.; Jiang, L. Effects of microwave on the structural and emulsifying properties and interfacial properties of oxidized soybean protein aggregates. Food Chem. X 2023, 19, 100861. https://doi.org/10.1016/j.fochx.2023.100861

S2. Zhao, Q.; Lin, J.; Wang, C.; Yousaf, L.; Xue, Y.; Shen, Q. Protein structural properties and proteomic analysis of rice during storage at different temperatures. Food Chem. 2021, 361, 130028. https://doi.org/10.1016/j.foodchem.2021.130028

S3. Sytar, O.; Bośko, P.; Živčák, M.; Brestic, M.; Smetanska, I. Bioactive phytochemicals and antioxidant properties of the grains and sprouts of colored wheat genotypes. *Molecules* **2018**, *23*, 2282. https://doi.org/10.3390/molecules23092282

S4. Plustea, L.; Dossa, S.; Dragomir, C.; Cocan, I.; Negrea, M.; Obistioiu, D.; Poiana, M.; Voica, D.; Berbecea, A.; Alexa, E. Comparative study of the nutritional, phytochemical, sensory characteristics and glycemic response of cookies enriched with lupin sprout flour and lupin green sprout. *Foods* **2024**, *13*, 656. https://doi.org/10.3390/foods13050656

S5. Dragomir, C.; Misca, C.D.; Dossa, S.; Jianu, C.; Radulov, I.; Negrea, M.; Paven, L.; Alexa, E. Chemical composition, antibacterial activity, and food application of sprouts from Fabaceae and Brassicaceae species. *Appl. Sci.* **2025**, *15*, 1896. https://doi.org/10.3390/app15041896

S6. Uivarasan, A.; Mihaly Cozmuta, L.; Peter, A.; Nicula, C.; Alexa, E.; Muresan-Pop, M.; Magyari, K.; Baia, M.; Szakács, Z.; Pintilii, C.; Vosgan, Z.; Mihalescu, B.; Mihaly Cozmuta, A. Microwave-Induced Modifications in Physicochemical Properties, Phenolic Compounds, and Color Stability of Whole Wheat and Black Wheat (*Triticum aestivum* L.) Flour. *J. Food Meas. Charact.* **2026**, *submitted.*

S7. Goñi, I.; Garcia-Alonso, A.; Saura-Calixto, F. A starch hydrolysis procedure to estimate glycemic index. Nutr. Res. 1997, 17(3), 427–437. https://doi.org/10.1016/S0271-5317(97)00010-9
